# A multifaceted molecular approach to surveillance of leishmaniasis: Identification of sand fly species, *Leishmania* parasites, and blood meal sources using high-resolution melting analysis

**DOI:** 10.1371/journal.pntd.0013412

**Published:** 2025-09-24

**Authors:** Liora Studentsky, Fouad Akad, Debora Diaz, Irina Ben Avi, Shirly Lea Elbaz, Tamar Grossman, Maya Davidovich-Cohen, Oscar David Kirstein, Laor Orshan, Gad Baneth

**Affiliations:** 1 Ministry of Health, Public Health Laboratories, Jerusalem, Israel; 2 The Hebrew University of Jerusalem, Rehovot, Israel; 3 Ministry of Health, National Laboratory for Public Health, Tel Aviv, Israel; Hokkaido University International Institute for Zoonosis Control, JAPAN

## Abstract

Leishmaniasis is a significant public health concern in large parts of the world including Israel, with limited diagnostic tools available for effective surveillance and control. Traditional methods for sand fly species identification, *Leishmania* detection, and blood meal analysis are time-consuming and prone to errors. To address these challenges, this study aimed to develop PCR-high resolution melt (HRM) assays to accurately determine sand fly species, *Leishmania* infection and blood meal sources in sand flies. Field-collected sand flies from all regions of Israel were used for the validation of three PCR-HRM assays. These included 254 sand fly males and females identified morphologically for species verification; 1,120 unfed females for *Leishmania* detection, and 538 engorged females for blood meal identification. PCR products were subjected to HRM curve analysis, and results were compared to nucleotide sequencing and sand fly morphology. Eleven sand fly species, 25 different host species blood meals and four *Leishmania* species were discerned and each presented a specific HRM pattern. Of the 1,658 analyzed females, 16 (1%) were positive for *Leishmania*, and the species identified were: *Leishmania major, L. tropica, L. infantum* and *L. donovani*. Blood meal source was identified in 520 (96.7%) engorged females. Blood from four animal species (domestic cat, rock hyrax, European hare, cow) accounted for 53% of the sand fly blood meals and the remaining 47% came from 21 other animal species. The sand fly species distribution showed that *L. major* and *L. donovani* vectors were mostly prevalent in arid southern Israel while *L. tropica* and *L. infantum* vectors were abundant in central and northern Israel. These results present the current knowledge of the different *Leishmania* species life cycles, vectors, and host species present in Israel and substantiate the utility of the assays developed herein which combine the advantages of PCR and the discriminatory power of HRM.

## Author summary

Leishmaniasis is an endemic disease in Israel and four *Leishmania* species are responsible for cutaneous or visceral leishmaniasis circulation in different zones of the country. This disease affects the health of humans and animals, such as dogs and cats. In this study we presented the development and validation of new analytic methods and conducted an entomological survey using Real-Time PCR and HRM analysis for sand fly and *Leishmania* species identification, and to determine blood meal sources of field-collected sand flies. The combination of these methods has elucidated the complex prevalence of different sand fly vectors, the most common sources of their blood meal, and the species of *Leishmania* parasites which they harbor, in Israel. This model would also be useful for epidemiological studies in other areas of the world where leishmaniasis is prevalent.

## Introduction

Leishmaniasis comprises a group of zoonotic vector-borne diseases caused by several species of *Leishmania* parasites. It is endemic in Israel, the Middle East, and many other parts of the world [1]. *Leishmania* species infect humans and animals and are transmitted by at least 98 confirmed and suspected sand fly vectors within the genera *Phlebotomus* and *Lutzomyia* [2]. Leishmaniasis is characterized by several remarkable paradoxes that challenge our understanding of its epidemiology and control. Although different *Leishmania* species can cause similar clinical symptoms [3–5], some species can induce a variety of clinical manifestations, such as *Leishmania infantum* causing visceral disease in some patients and cutaneous disease in others [6–8].

At least 70 species of wild and domestic animals have been identified as confirmed or potential reservoirs involved in *Leishmania* transmission cycles [9,10]. Cutaneous leishmaniasis (CL), alongside visceral leishmaniasis (VL), are notifiable diseases with significant public health implications in Israel. The number of new cases reported annually in Israel is shown in S1 Fig [11–15]. Analysis of disease incidence reveals a concerning trend. Between 2000 and 2004, an average of 58 CL cases were reported annually. This number rose sharply to approximately 200 cases per year between 2005 and 2009. Data from 2010-2014 shows a further surge, with an average of 304 cases reported annually. A peak in the number of cases was observed in 2012–2014, with disease emerging in previously unaffected areas [16,17]. In the following five years, 2015–2019, a decrease was observed in human morbidity, with an average of 237 CL and 5.4 VL annual cases. A further decrease occurred in human morbidity in 2020–2023, with an average 127 CL and one VL cases reported annually [11].

Leishmaniasis in Israel presents complex zoonotic transmission patterns involving four *Leishmania* species: *Leishmania major*, *Leishmania tropica*, *L. infantum*, and *Leishmania donovani*, leading to a variety of clinical manifestations in humans and mammals across different ecoregions of the country. All four species cause CL, however, *L. infantum* is responsible mainly for VL [12–19]. Each *Leishmania* species present in Israel has a distinct transmission cycle involving specific reservoir hosts. *Leishmania major* is transmitted by *Phlebotomus papatasi* with a diverse range of vertebrate reservoir hosts, including the fat sand rats (*Psammomys obesus*), gerbils (*Gerbillus dasyurus*), jirds (*Meriones crassus* and *Meriones tristrami*), and voles (*Microtus guentheri*). *Leishmania tropica*, transmitted by *Phlebotomus sergenti* and *Phlebotomus arabicus*, is associated with rock hyraxes (*Procavia capensis*) as a primary reservoir host [20–23]. Putative vectors for *L. infantum* include *Phlebotomus perfiliewi galilaeus*, *Phlebotomus syriacus*, and *Phlebotomus tobbi*, with various canid species serving as reservoir hosts [12]. A fourth CL transmission cycle in southern Israel involves *L. donovani* possibly transmitted by *Phlebotomus alexandri* and maintained by hares (*Lepus europaeus*) as reservoir hosts [19,24].

High-resolution melting (HRM) PCR has emerged as a promising approach that offers several advantages over traditional molecular methods. HRM-PCR utilizes the thermal denaturation characteristics of amplicons. The melting temperature (Tm) and typical melting curve shape deduced by the HRM-PCR, are determined by nucleotide sequences, GC content, and amplicon length, and provide valuable information for species-specific genotype identification, making it a relatively cost-effective method compared to traditional PCR techniques [25–28].

This study aimed to develop new HRM-PCR-based assays for identification of sand fly and *Leishmania* species, and blood meal sources of sand flies as tools for surveillance and control of leishmaniasis.

## Materials and methods

### Sand fly field collection

Sand flies were collected in entomological surveys conducted by the medical entomology laboratory of the Israeli Ministry of Health between 2022 and 2023 in areas of Israel known for the existence of different *Leishmania* transmission cycles. The surveys encompassed geographically diverse areas, including the Sea of Galilee region, central Israel, the Jerusalem area, southeastern Israel, and the Arava and Negev deserts. Modified CDC (Centers for Disease Control) traps baited with dry ice, were employed for overnight outdoor collections. The use of CO₂ alone, without a light source, follows established national protocols and is based on evidence showing light traps are ineffective for sand fly capture in Israel [29]. The traps were placed on updraft position with their opening placed ~ 1m above the ground, following established protocols [29,30].

### Sand fly sample preparation

Collected sand flies were sexed and identified for species level at the medical entomology laboratory at the Israeli Ministry of Health in Jerusalem. Male specimens were morphologically characterized to the species level based on genitalia features. Individual, unfed female sand flies were dissected to examine spermathecal and pharyngeal morphology following established identification keys [31,32]. Individual female sand fly abdomens were separated from the rest of the body using forceps on a chilled plate. Forceps were cleaned with 10% bleach and water between dissections to avoid cross-contamination. Each abdomen was placed in an individual 1.1 ml microcentrifuge tube and stored at -80°C for subsequent DNA extraction and molecular analyses.

### DNA extraction

Total genomic DNA was extracted from sand fly samples and from *Leishmania* parasite promastigote cultures of national and international reference strains obtained from the Kuvin Center cryobank (Hebrew University of Jerusalem) including *L. major* (MHOM/PS/1967/JerichoII), *L. tropica* (MHOM/IL/1990/P283), *L. infantum* (MHOM/SD/62/2S), *L. donovani* (MHOM/SD/1962/1S-CLD2) and *Leishmania aethiopica* (MHOM/ET/1972/L102).

The DNA extraction protocol involved homogenization in 50 μL in-house lysis buffer containing DNase- and proteinase-free RNaseA (Thermo Fisher Scientific, Cat. No. EN0531), proteinase K, and ATL tissue lysis buffer (QIAGEN, Cat. No./ID:19076). Sand fly tissues were disrupted using the TissueLyser II (QIAGEN) with 3-mm stainless-steel beads for 5 minutes. Following homogenization, an additional 200 μL of the same lysis buffer was added to each sample and incubated at 56°C for two hours. Subsequently, homogenates were centrifuged, and total DNA was extracted using the QIAsymphony DSP DNA Mini Kit (192) (QIAGEN, Cat. No./ID:937236) in a QIAGEN’s QIAsymphony SP instrument (Cat. No./ID:9001297) following the manufacturer’s instructions. Purified DNA was eluted in 100 μL of kit elution buffer.

### Real-time PCR and HRM analysis

Three separate real-time PCR reactions targeting sand fly species identification, *Leishmania* species detection and identification, and blood meal source analysis, were carried out using a Roche LightCycler 96 instrument (Roche, Mannheim, Germany) with AccuMelt HRM SuperMix (Quanta Bioscience, Gaithersburg, USA, Cat. no. 95103–012) and specific primers (Table 1) following protocols detailed in Studentsky et al. 2023 [19]. HRM analysis was performed following the real-time PCR amplification. Amplicon dissociation analysis was carried out by capturing fluorescence signals in 0.1°C/sec intervals and holding for 60 seconds in each range of the melting curve (between 60°C and 85°C, in the sand fly species and blood meal detection assays, or up to 95°C in the case of *Leishmania* PCR). Genotype assignment was based on reference controls representing known species, allowing for the recognition of unknown samples based on their melting peak profiles. Normalized melting curves and peak profiles with distinct HRM profiles were categorized into groups based on identical melting peak patterns observed in all individuals of the same species.

**Table 1:** Real-time PCR primers used in the study.

Reaction	Target gene	Primers names	Primers sequences	Annealing temp. (◦C)	Product size	Ref.
***Leishmania* species**	Internal transcribed spacer 1 locus (ITS1)	ITS1-219F	5’-AGCTGGATCATTTTCCGATG-3’	60°C	265 bp	[33]
		ITS1-219R	5’-ATCGCGACACGTTATGTGAG-3’			
**Sand fly species**	Cytochrome b gene (*cytb*)	cytb-F	5’-GGAGGAGTAATYGCHYTTGTWATATC-3’	60°C	368-393 bp	[19]
		cytb-R	5’-AAGATATTTACCYGCTTCKTTATGTT-3’			
**Blood meal source**	*12S* and *16S* genes	N12-16F	5’-ACAYACCGCCCGTCACCCTC-3’	61°C	500 bp	[19]
		N12-16R	5’-AACCAGCTATCACMAGGCTCG-3’			

### HRM-based sand fly species identification

Validation of HRM-PCR was conducted on morphologically identified sand flies (n = 254, 131 males and 123 females). This assay targeted a 368–393-bp fragment of the *cytochrome b* gene (cytb) for *Phlebotomus* species identification (Table 1). The established HRM profiles established in this study include all twelve sand fly species documented in Israel to date (Table 2). Following HRM analysis, samples representing distinct melting peak groups were sequenced for confirmation.

**Table 2:** Sand fly species and the number of specimens identified using both morphological examination and High-Resolution Melting (HRM) analysis.

Sand fly species	Number of samples analyzed		Total sand fly number
	Males	Females	
***Phlebotomus alexandri***	15	12	27
***Phlebotomus arabicus***	6	11	17
***Phlebotomus canaaniticus***	5	4	9
***Phlebotomus halepensis***	11	6	17
***Phlebotomus jacusieli***	5	6	11
***Phlebotomus kazeruni***	14	15	29
***Phlebotomus papatasi***	16	13	29
***Phlebotomus perfiliewi galilaeus***	15	11	26
***Phlebotomus sergenti***	14	11	25
***Phlebotomus simici***	5	11	16
***Phlebotomus syriacus***	15	11	26
***Phlebotomus tobbi***	13	9	22
**Total**	**131**	**123**	**254**

### *Leishmania* detection and identification

*Leishmania* species HRM profiles validation was conducted using two types of controls: (1) parasite promastigote cultures of international reference strains of *Leishmania,* (*L. major* MHOM/PS/1967/JerichoII, *L. tropica* MHOM/IL/1990/P283, *L. infantum* MHOM/SD/62/2S, *L. donovani* MHOM/SD/1962/1S-CLD2 and *L. aethiopica* MHOM/ET/1972/L102) and, (2) *Leishmania* American Type Culture Collection (ATCC) controls containing genomic DNA from *L. major* (Cat. no. 30012D), *L. tropica* (Cat. no. 50129D), *L. infantum* (strain MHOM/TN/80IPT-1, Cat. no. 50134D) and *L. donovani* (strain Khartoum, Cat. no. 30030D). Molecular biology-grade water (Bio-Lab, Israel) served as a negative control (NC).

### Sand fly blood meal source identification

The HRM method for blood meal source identification was validated using 38 DNA samples from 13 different vertebrate species provided by the Koret School of Veterinary Medicine, Hebrew University (Table 3). A human DNA (*Homo sapiens*) sample, provided by the Parasitology National Reference Center, Israel Ministry of Health, was included in the tests as a part of their diagnostic procedure. Blood meal identification in field-collected engorged sand flies (n = 538) was performed by amplifying a 500-bp DNA fragment of the host 12S and 16S mitochondrial *rRNA* genes using universal vertebrate primers [19] (Table 1).

**Table 3:** Species of vertebrates used as positive controls for sand fly blood meal identification.

Species name	Common name	Number of samples	Source
***Bos Taurus***	Cow	1	Blood
***Canis aureus***	Jackal	1	Blood
***Canis lupus***	Wolf	1	Blood
***Canis lupus familiaris***	Dog	1	Blood
***Equus cabalus***	Horse	1	Blood
***Felis catus***	Cat	4	Blood
***Homo sapiens***	Human	1	Blood
***Lepus europaeus***	Hare	8	Tissue
***Meles meles***	Badger	1	Blood
***Meriones tristrami***	Jird	7	Tissue
***Procavia capensis***	Rock hyrax	1	Blood
***Psammomys obesus***	Sand rat	10	Blood
***Vulpes vulpes***	Fox	1	Blood

### Sample sequencing and sequence alignment

Following HRM analyses, all samples categorized into distinct melting curve groups underwent Sanger sequencing at the Center for Genomic Technologies (Hebrew University) for confirmation. Sequence alignment and correction were conducted using BioNumerics v8.0 software (Applied Maths, Belgium) and compared with sequences in the GenBank database via the NCBI BLASTn algorithm (http://blast.ncbi.nlm.nih.gov/Blast.cgi). *Leishmania* species, blood meal sources, and sand fly species were identified when their sequences were the top BLAST match and exhibited ≥98% identity.

## Results

A total of 1,912 sand flies from the entomological survey were used, including 131 males and 1,781 females (1,243 unfed and 538 fed).

### 1. Development and validation of the HRM assays

#### Sand fly species identification HRM assay.

**Fig 1:**
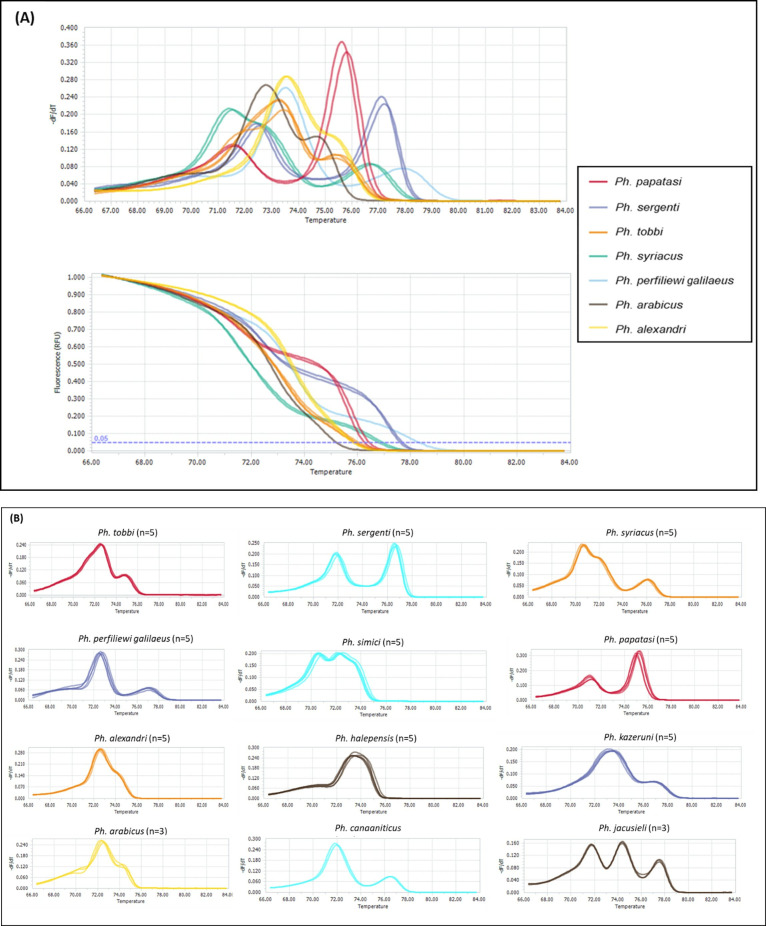
Melting peaks and curves of different sand fly species. **(A)** Combined normalized melting peaks (top) and curves (bottom) of sand fly species using the HRM-RT-PCR analysis of the *Cyt b* gene; **(B)** The melting peaks of each separate sand fly species *Cyt b* gene sequences.

To evaluate the reliability of the HRM analysis for sand fly species identification, a total of 254 sand flies (131 males and 123 unfed females) were initially morphologically identified to species level. Subsequently, their DNA was extracted and subjected to HRM analysis targeting a fragment of the *cytb* gene (368–393 bp). Each sample was analyzed using three methods: morphological identification, HRM, and Sanger sequencing (Tables 4, S1, and S1 File). The HRM analysis generated a unique “fingerprint” pattern for each sand fly species characterized by a specific melting temperature (Tm peak). Furthermore, the HRM results were able to group the tested species into 12 distinct patterns. Each species exhibited a characteristic melting peak pattern, enabling clear differentiation among sand fly species (Fig 1). Notably, 12 (4.7%) out of 254 samples initially misidentified by morphological features were correctly identified by HRM and confirmed through DNA sequencing (Tables 4, S1).

**Table 4:** Male (M) and unfed female (F) sand fly sample identification by morphology, HRM-Real-Time PCR and sequencing.

Sand fly species	Sand fly number	Morphological identification	HRM RT-PCR identification	DNA sequence identification
***Phlebotomus alexandri***	27 (13 M, 14 F)	*Ph. alexandri*	*Ph. alexandri*	*Ph. alexandri*
***Phlebotomus arabicus***	14 (7 M, 7 F)	*Ph. arabicus*	*Ph. arabicus*	*Ph. arabicus*
	2 (2 M)	*Ph. simici*	*Ph. arabicus*	*Ph. arabicus*
	1 (1 M)	*Ph. halepensis*	*Ph. arabicus*	*Ph. arabicus*
***Phlebotomus canaaniticus***	9 (4 M, 5 F)	*Ph. canaaniticus*	*Ph. canaaniticus*	*Ph. canaaniticus*
***Phlebotomus halepensis***	16 (7 M, 9 F)	*Ph. halepensis*	*Ph. halepensis*	*Ph. halepensis*
	1 (1 F)	*Ph. sergenti*	*Ph. halepensis*	*Ph. halepensis*
***Phlebotomus jacusieli***	10 (4 M, 6 F)	*Ph. jacusieli*	*Ph. jacusieli*	*Ph. jacusieli*
	1 (1 M)	*Paraphlebotomus sp.*	*Ph. jacusieli*	*Ph. jacusieli*
***Phlebotomus kazeruni***	29 (18 M, 11 F)	*Ph. kazeruni*	*Ph. kazeruni*	*Ph. kazeruni*
***Phlebotomus papatasi***	29 (16 M, 13 F)	*Ph. papatasi*	*Ph. papatasi*	*Ph. papatasi*
***Phlebotomus perfiliewi galilaeus***	25 (12 M, 13 F)	*Ph. perfiliewi galilaeus*	*Ph. perfiliewi galilaeus*	*Ph. perfiliewi galilaeus*
	1 (1 M)	*Ph. syriacus*	*Ph. perfiliewi galilaeus*	*Ph. perfiliewi galilaeus*
***Phlebotomus sergenti***	23 (9 M, 14 F)	*Ph. sergenti*	*Ph. sergenti*	*Ph. sergenti*
	1 (1 M)	*Ph. alexandri*	*Ph. sergenti*	*Ph. sergenti*
	1 (1 M)	*Ph. jacusieli*	*Ph. sergenti*	*Ph. sergenti*
***Phlebotomus simici***	13 (5 M, 8 F)	*Ph. simici*	*Ph. simici*	*Ph. simici*
	2 (1 M, 1 F)	*Ph. syriacus*	*Ph. simici*	*Ph. simici*
	1 (1 M)	*Ph. canaaniticus*	*Ph. simici*	*Ph. simici*
***Phlebotomus syriacus***	26 (11 M, 15 F)	*Ph. syriacus*	*Ph. syriacus*	*Ph. syriacus*
***Phlebotomus tobbi***	21 (10 M, 11 F)	*Ph. tobbi*	*Ph. tobbi*	*Ph. tobbi*
	1 (1 M)	*Ph. perfiliewi galilaeus*	*Ph. tobbi*	*Ph. tobbi*

#### HRM assay for detection and differentiation of *Leishmania* species in sand flies.

**Fig 2:**
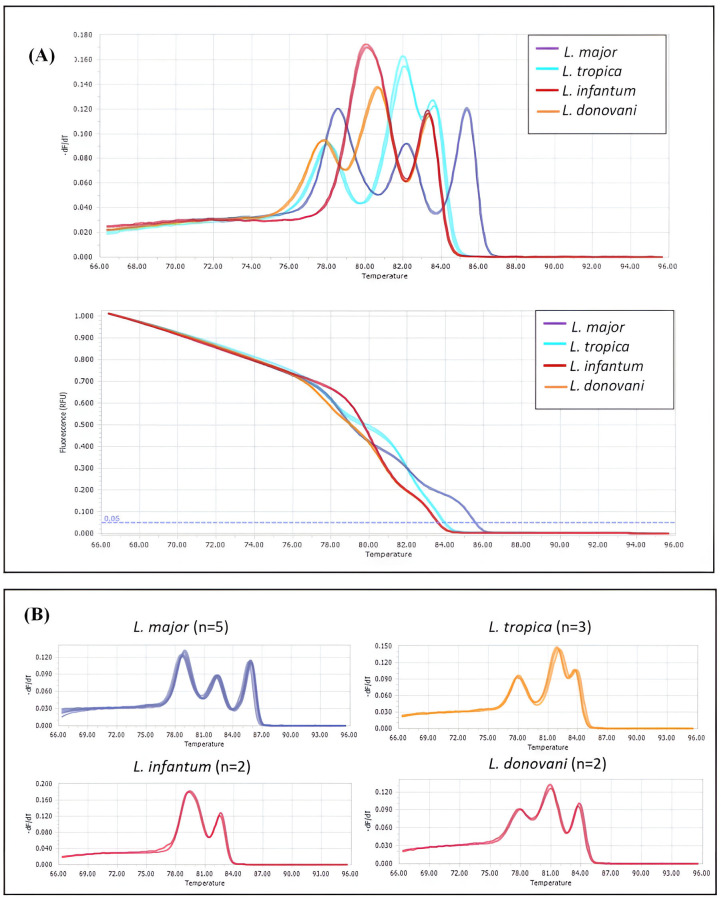
Melting peaks and curves of different *Leishmania* species. **(A)** Combined normalized melting peaks (top) and curves (bottom) of *Leishmania* species using the HRM-RT-PCR analysis of ITS1 locus; **(B)** The melting peaks of separate *Leishmania* species ITS1 sequences.

For *Leishmania* species identification, HRM analysis targeting the internal transcribed spacer 1 (ITS1) locus (~265 bp) [33], was validated using DNA from reference *Leishmania* cultures (*L. major, L. tropica, L. infantum*, and *L. donovani*) and ATCC controls to characterize specific HRM profiles for each species based on unique melting peaks (Fig 2). These profiles were then used to identify *Leishmania* DNA in field-collected sand flies.

The HRM peaks for *L. infantum* exhibited an HRM curve with two prominent peaks, whereas the other three *Leishmania* species found in Israel produced three peaks. *Leishmania major* exhibited three peaks, with the middle peak being the lowest. In contrast, *L. donovani* had a similar peak pattern but with the middle peak being the highest. *Leishmania tropica* was characterized by three peaks, with a smaller peak on the right (Figs 2, S1 File).

#### Sand flies blood meal identification HRM assay.

**Fig 3:**
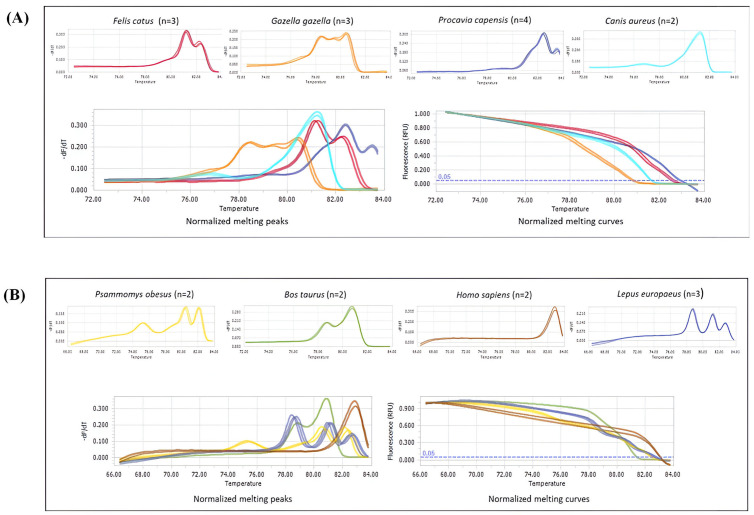
Melting peaks and curves of different sand fly blood meal sources. Separate (top) and combined (bottom) normalized melting peaks and curves of blood meal sources of sand flies found in this study using the HRM-RT-PCR analysis of *12S*, *16S rRNA* genes; **(A)** *Felis catus* (domestic cat), *Gazella gazella* (mountain gazelle), *Procavia capensis* (rock hyrax), *Canis aureus* (golden jackal); **(B)** *Psammomys obesus* (fat sand rat), *Bos taurus* (cow), *Homo sapiens* (human), *Lepus europaeus* (European hare).

An HRM assay targeting a fragment of the vertebrate *12S* and *16S* mitochondrial *rRNA* genes (~500 bp) was developed and validated to identify blood meal sources of sand flies. DNA from various relevant vertebrate hosts and potential hosts (fat sand rats, European hares, domestic cats, domestic dogs, and humans) was used to generate a reference library of unique HRM profiles for each species (Fig 3). The figure depicts some of the detected blood sources. Each blood source exhibited a distinct HRM pattern. In cases when HRM patterns had similarities, the PCR Tm at the beginning of the peaks was different and enabled to reliably differentiate between the types of blood sources. For example, the golden jackal (*Canis aureus*) (Fig 3A) and the human (*Homo sapiens*) exhibited single major peak patterns. However, their Tm differed, with the golden jackal at 78°C, preceded by a small curve, and the human at 81°C. The domestic cat (*Felis catus*), cow (*Bos taurus*), and rock hyrax (*Procavia capensis*) produced curves that had two prominent peaks, but created very different patterns which could not be confused (Fig 3). In addition, although the European hare (*Lepus europaeus*), fat sand rat (*Psammomys obesus*) and mountain gazelle (*Gazella gazella*) produced HRM patterns with three peaks, those patterns were different from each other with the fat sand rat creating an initial low peak and then two higher peaks, the European hare creating three prominent peaks toward the end of the pattern, and the gazelle having three consecutive peaks with similar heights that almost create a plateau space between them (Figs 3, S1 File).

### 2. HRM analysis of field-collected sand flies

HRM analysis for *Leishmania* species identification was conducted on 1,120 unfed females sand flies, which were screened for *Leishmania* DNA and parasite species identification (Table 1) [27,33]. Positive samples were further confirmed by DNA sequencing. Ten unfed sand fly females were found infected with a *Leishmania* sp. and all positive samples were classified by HRM-RT-PCR and correctly verified by DNA sequencing into four *Leishmania* species (Table 5). The HRM melting peaks of all 1,120 trapped unfed female sand flies matched the HRM profiles groups of the reference species (Fig 1). Eleven sand fly species were identified and only three of them, *Ph. sergenti*, *Ph. syriacus* and *Ph. alexandri* were found infected with *L. tropica*, *L. infantum,* or *L. donovani* parasites, respectively (Table 5). *Phlebotomus canaaniticus*, the twelfth species for which the unique HRM profiles was determined in sand fly species validation, was not detected among the analyzed field-collected females.

**Table 5:** Sand fly species and *Leishmania* parasites infection detected by HRM analysis of collected unfed and fed sand fly females.

Sand fly species	Unfed/Fed	Sand fly number	*Leishmania* species			
			*L. major*	*L. tropica*	*L. infantum*	*L. donovani*
***Ph. Papatasi***	Unfed	219	0	0	0	0
	Fed	119	**2**	0	0	0
***Ph. Sergenti***	Unfed	216	0	**6**	0	0
	Fed	199	0	**1**	0	0
***Ph. Syriacus***	Unfed	182	0	0	**1**	0
	Fed	33	0	0	0	0
***Ph. alexandri***	Unfed	82	0	0	0	**3**
	Fed	102	0	0	0	**3**
***Ph. arabicus***	Unfed	2	0	0	0	0
	Fed	3	0	0	0	0
***Ph. halepensis***	Unfed	11	0	0	0	0
	Fed	2	0	0	0	0
***Ph. Jacusieli***	Unfed	1	0	0	0	0
	Fed	0	0	0	0	0
***Ph. kazeruni***	Unfed	48	0	0	0	0
	Fed	4	0	0	0	0
***Ph. perfiliewi galilaeus***	Unfed	101	0	0	0	0
	Fed	33	0	0	0	0
***Ph. Simici***	Unfed	2	0	0	0	0
	Fed	0	0	0	0	0
***Ph. Tobbi***	Unfed	256	0	0	0	0
	Fed	43	0	0	0	0
**Total sand fly number**	**Unfed**	**1,120**	**0**	**6**	**1**	**3**
	**Fed**	**538**	**2**	**1**	**0**	**3**

In addition, ten different sand fly species were identified among the 538 blood-fed sand fly females which were analyzed using the HRM assay to identify sand fly species and *Leishmania* infection (Table 5). Six females of *Ph. papatasi, Ph. sergenti* and *Ph. alexandri* were identified as harboring the following *Leishmania* parasites: *L. major*, *L. tropica* and *L. donovani,* respectively (Table 5).

**Fig 4:**
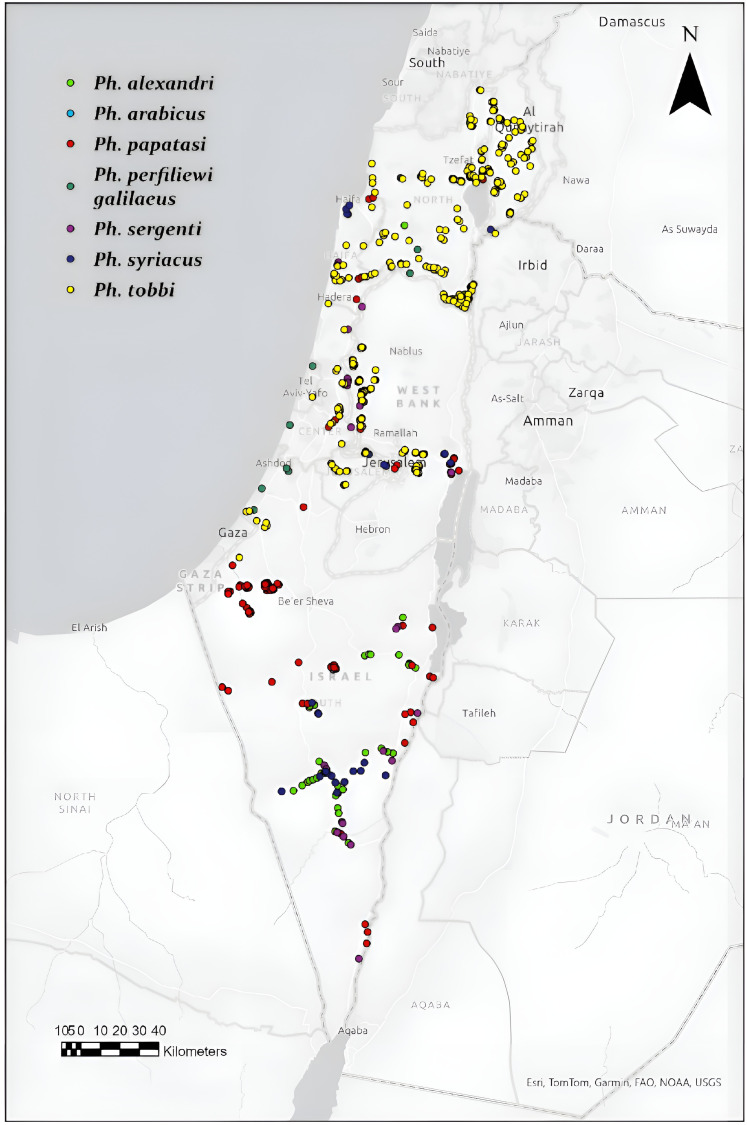
Distribution of proven and potential phlebotomine sand fly vectors of *Leishmania* species identified in this study. Map was created using ArcGIS software. Base map source: Natural Earth (https://www.naturalearthdata.com/), public domain.

The locations in Israel where different sand fly species were detected in the study are shown in Fig 4, where proven vectors and putative vectors of *Leishmania* species were included, and in S2 Fig where phlebotomine species which are not known vectors are shown. *Phlebotomus papatasi*, the vector of *L. major* was found in large quantities in the south and only in smaller numbers in the central parts of Israel. *Phlebotomus alexandri,* known as a vector of *L. donovani* in Israel, was found mostly in the Negev desert of southern of Israel and in few locations in other parts of the country. *Phlebotomus sergenti*, the main vector of *L. tropica* in Israel was distributed from the Dead Sea to northern Israel, and *Ph. arabicus* which is also a vector of *L. tropica* was found only in northern Israel. The putative vectors of *L. infantum,* the sand fly species *Ph. syriacus*, *Ph. tobbi* and *Ph. perfiliewi galilaeus*, were found predominantly in central and northern Israel (Fig 4).

Additional sand fly species which are not known as *Leishmania* vectors, were also trapped and identified. *Phlebotomus kazeruni*, was found in the Negev desert in southern Israel. *Phlebotomus jacusieli* is a relatively rare species which was trapped near Jerusalem and in other parts of central Israel. Other non-vector species including *Phlebotomus halepensis*, *Phlebotomus simici* and *Ph. canaaniticus* were distributed near the Sea of Galilee and in other parts of northern Israel (Fig 4).

HRM-based blood meal identification was successful in 96.7% (n = 520) of the blood–fed females tested. In addition to known blood meal sources which could be identified by matching to sequences in GenBank, previously unknown blood meal sources were identified through sequencing. Sequence analysis revealed a total of 25 different blood meal sources, with blood from humans, domestic animals, birds, rodents, and other wild animals, including several identified vertebrates known as *Leishmania* reservoirs (dogs, jackals, rock hyraxes, jirds, hares) (Table 6, Fig 3) [10,19,22,34,35]. Only nine of the eleven field-collected sand fly species included engorged females. No blood-fed *Ph. jacusieli* and *Ph. simici* were collected.

**Table 6:** Sources of sand fly blood meals detected in the study. Details of each sand fly species associated with every detected blood source are provided, along with their respective GenBank accession numbers.

Blood meal source species name (number)	Common name	GenBank accession number	Sand fly species in which the blood meal was found	Proportion of certain blood meal in particular sand fly species
***Alectoris chukar*** (n= = 3)	Chukar partridge	PQ622922	*Ph. sergenti*	3/3 (100%)
***Bos taurus*** (n= = 57)	Cow	PQ852113	*Ph. perfiliewi galilaeus*	22/57 (39%)
			*Ph. papatasi*	14/57 (25%)
			*Ph. syriacus*	12/57 (21%)
			*Ph. tobbi*	8/57 (14%)
			*Ph. sergenti*	1/57 (2%)
***Canis aureus*** (n= = 9)	Golden jackal	PQ622917	*Ph. perfiliewi galilaeus*	4/9 (44%)
			*Ph. tobbi*	3/9 (33%)
			*Ph. papatasi*	2/9 (22%)
***Canis lupus familiaris*** (n= = 32)	Domestic dog	PQ622923	*Ph. sergenti*	12/32 (37.5%)
			*Ph. tobbi*	7/32 (22%)
			*Ph. papatasi*	4/32 (12.5%)
			*Ph. syriacus*	3/32 (9%)
			*Ph. alexandri*	2/32 (6%)
			*Ph. halepensis*	2/32 (6%)
			*Ph. perfiliewi galilaeus*	2/32 (6%)
***Capra hircus*** (n= = 17)	Goat	PQ622921	*Ph. perfiliewi galilaeus*	9/17 (53%)
			*Ph. tobbi*	6/17 (35%)
			*Ph. papatasi*	1/17 (6%)
			*Ph. sergenti*	1/17 (6%)
***Columba livia*** (n= = 1)	Rock dove	PQ622926	*Ph. sergenti*	1/1 (100%)
***Equus asinus*** (n= = 5)	Donkey	PQ622916	*Ph. kazeruni*	4/5 (80%)
			*Ph. sergenti*	1/5 (20%)
***Equus caballus*** (n= = 7)	Horse	PQ622919	*Ph. papatasi*	5/7 (71%)
			*Ph. tobbi*	2/7 (29%)
***Equus hemionus*** (n= = 30)	Onager	PQ622939	*Ph. alexandri*	27/30 (90%)
			*Ph. kazeruni*	2/30 (7%)
			*Ph. sergenti*	1/30 (3%)
***Erinaceus concolor*** (n= = 10)	Hedgehog	PQ622925	*Ph. papatasi*	10/10 (100%)
***Felis catus*** (n= = 89)	Domestic cat	PQ852112	*Ph. sergenti*	76/89 (85%)
			*Ph. tobbi*	7/89 (8%)
			*Ph. papatasi*	4/89 (4.5%)
			*Ph. syriacus*	2/89 (2.5%)
***Gazella dorcas*** (n= = 10)	Dorcas gazelle	PQ622928	*Ph. alexandri*	10/10 (100%)
***Gazella gazella*** (n= = 8)	Mountain gazelle	PQ622931	*Ph. sergenti*	6/8 (75%)
			*Ph. syriacus*	1/8 (12.5%)
			*Ph. tobbi*	1/8 (12.5%)
***Hemiechinus auritus*** (n= = 4)	Long-eared hedgehog	PQ622924	*Ph. papatasi*	4/4 (100%)
***Homo sapiens*** (n= = 4)	Human	PQ622920	*Ph. papatasi*	2/4 (50%)
			*Ph. alexandri*	2/4 (50%)
***Hystrix indica*** (n= = 4)	Indian crested porcupine	PQ622934	*Ph. sergenti*	4/4 (100%)
***Lepus europaeus*** (n= = 63)	European hare	PQ622935	*Ph. alexandri*	53/63 (84%)
			*Ph. papatasi*	5/63 (8%)
			*Ph. sergenti*	3/63 (5%)
			*Ph. kazeruni*	1/63 (2%)
			*Ph. tobbi*	1/63 (2%)
***Meles meles*** (n= = 1)	European badger	PQ622937	*Ph. papatasi*	1/1 (100%)
***Meriones tristram***i (n= = 25)	Tristram’s jird	PQ622918	*Ph. papatasi*	25/25 (100%)
***Mus musculus*** (n= = 3)	House mouse	PQ622938	*Ph. papatasi*	3/3 (100%)
***Ovis aries*** (n= = 22)	Domestic sheep	PQ852114	*Ph. papatasi*	9/22 (41%)
			*Ph. sergenti*	7/22 (32%)
			*Ph. perfiliewi galilaeus*	3/22 (14%)
			*Ph. tobbi*	2/22 (9%)
			*Ph. halepensis*	1/22 (6%)
***Procavia capensis*** (n= = 75)	Rock hyrax	PQ622930	*Ph. sergenti*	66/75 (88%)
			*Ph. papatasi*	7/75 (9%)
			*Ph. arabicus*	2/75 (3%)
***Psammomys obesus*** (n= = 6)	Fat sand rat	PQ622940	*Ph. papatasi*	6/6 (100%)
***Sus scrofa*** (n= = 15)	Wild boar	PQ622929	*Ph. tobbi*	7/15 (47%)
			*Ph. papatasi*	4/15 (27%)
			*Ph. sergenti*	2/15 (13%)
			*Ph. syriacus*	2/15 (13%)
***Vulpes vulpes*** (n= = 20)	Red fox	PQ622927	*Ph. sergenti*	14/20 (70%)
			*Ph. papatasi*	4/20 (20%)
			*Ph. alexandri*	2/20 (10%)

The wide variety of blood meals found in the different sand fly species (Tables 6, S2), provides an interesting insight into the feeding habits of sand flies in Israel. The most frequently detected blood meal sources were domestic cat (89/538; 16.5%), rock hyrax (75/538; 13.9%), European hare (63/538; 11.7%), and cow (57/538; 10.6%). Altogether these four animal species accounted for almost 52.9% of all blood meals while 21 other species accounted for 43.8% of the blood meals and an additional 3.3% could not be identified. In addition to this, it is also clear that a variety of sand fly species fed on each of the animal blood source species, with eight different species that have fed on dogs, six on cows, five on hares, and four on cats (Table 6). Despite this, *Leishmania* species DNA was found only in four of eleven sand fly species. (Tables 5, S2), and in the sand fly species known to be a vector for each of these *Leishmania* species in Israel and the Middle East. Accordingly, *L. major* DNA was found in *Ph. papatasi* fed on Tristram’s jird, *L. tropica* in *Ph. sergenti* which fed on rock hyraxes, and *L. donovani* in *Ph. alexandri* that fed on European hares [12,19,22].

## Discussion

This study describes the development and validation of HRM-PCR assays as a robust tool for sand fly species identification, detection of *Leishmania* infection, and blood meal sources characterization in field-collected sand flies. The HRM has previously been used to identify different species of *Leishmania* in human leishmaniasis patients, naturally infected dogs, and other animal hosts [33]. The HRM assay is a rapid, closed-tube system with high sensitivity, eliminating the need for laborious post-PCR processing steps and reducing the risk of contamination inherent in manipulation techniques. The findings demonstrate the efficacy of HRM analysis in overcoming the limitations of traditional morphological identification and Sanger sequencing, providing an accurate, rapid, and cost-effective method for entomological and epidemiological studies related to leishmaniasis and potentially other sand fly-borne diseases. In addition, unlike Sanger sequencing, restriction fragmentation length polymorphism (RFLP), or serological techniques, the HRM method provides results in under two hours without requiring specialized post-PCR steps or advanced bioinformatics, making it particularly suitable for routine diagnostics and large-scale field studies.

We developed, adapted, and tested the surveillance method on 1,781 female sand flies from different regions of Israel, identifying four *Leishmania* species, each with a unique melting peak pattern. The HRM assay identified twelve sand fly species, each exhibiting a unique “fingerprint” pattern with species-specific melting peaks. The ability of the HRM assay to differentiate species is particularly significant, as 4.7% (9 out of 254) of the sand flies were initially misidentified morphologically but were correctly identified by HRM and confirmed by DNA sequencing. This not only underscores the potential for morphological misidentification but also highlights the reliability of the assay as a diagnostic tool, especially in regions with high sand fly species diversity or cryptic species complexes. In some cases, HRM profiles exhibited multiple melting peaks from a single PCR amplicon. This may result from intra-species sequence variability or the presence of secondary structures especially within the ITS1 region. These observations are consistent with previously reported HRM data and do not affect diagnostic accuracy, as the dominant Tm remains species-specific and stable. The Tm values obtained in our HRM assays (S3 Table) are consistent with those reported in previous studies targeting similar loci. For *Leishmania* spp., Talmi-Frank et al. [22] described comparable ITS1-based melting profiles, supporting the robustness of this approach for species differentiation. Regarding vertebrate blood meal sources, the HRM profiles are widely used for blood identification observed in our study as well as other studies, substantiating the reliability of the method for host identification [19,36,37]. Although published literature on HRM-based identification of sand fly species remains limited, our findings demonstrate the accuracy, reproducibility, and discriminatory power of this method. These results provide strong support for the utility of HRM-PCR as a reliable tool for sand fly species identification and highlight its potential for broader application in future entomological studies.

While mitochondrial markers such as the *cytb* gene are widely used for molecular identification, we acknowledge that introgression or retention of ancestral polymorphism may rarely lead to misidentification of closely related species.

Identifying the blood meal sources in hematophagous vectors is crucial for monitoring and managing potential disease transmission foci [38]. Blood meals from 25 different animal host species, as well as human blood, were detected in 538 blood-fed female sand flies. The blood meal could be identified in 520 sand flies and only 18 (3.3%) were not be amplified by HRM PCR and were therefore not be identified molecularly. All three molecular targets (sand fly spp., *Leishmania* spp., and blood meal sources) can be discerned from a single sand fly specimen. Total DNA extracted from whole individual sand flies is suitable for parallel HRM-PCR assays targeting the *cytb* (for sand fly), *12S* and *16S* (for host blood meal) and ITS1 (for *Leishmania*). This approach maximizes data yield from each specimen and supports integrated surveillance and vector incrimination efforts.

*Leishmania* amastigotes acquired during a blood meal can develop into promastigotes and multiply within the blood bolus. However, if they are ingested by a non-vector sand fly species, they are typically expelled during blood defecation. For this reason, the mere identification of *Leishmania* parasites or their DNA in engorged sand fly females (before defecation) is insufficient for vector incrimination [39–41]. However, the detection of *L. tropica* in unfed *Ph. sergenti*, *L. infantum* in unfed *Ph. syriacus*, and *L. donovani* in unfed *Ph. alexandri*, provides further evidence supporting their possible role as active vectors of these *Leishmania* species.

Although the primary aim of this study was to establish and validate diagnostic PCR techniques for identifying sand fly species, blood meal sources and *Leishmania* infection, it did not involve random sampling of sand flies across the entire country. However, it did focus on four geographically diverse regions of Israel where both cutaneous and visceral leishmaniasis circulate. The outcomes of this study, identifying the species of sand flies found in each geographical area (Figs 4), determining the most frequent animal hosts for blood meal sources, and detecting the species of *Leishmania* present in particular sand fly species, provide valuable information regarding the epidemiology of leishmaniasis in Israel. HRM analysis distinguished all the Old World *Leishmania* species causing disease in Israel and its neighboring countries, including *L. infantum* and *L. donovani* as previously reported in other studies [35,42,43]. These two species both belong to the *L. donovani* complex and can be distinguished based on a short, species-specific DNA sequence in the ITS1 locus [19].

The diversity and distribution of sand fly species in Israel, as demonstrated in this study, are complex and are most likely influenced by the diversity of ecoregions across the country. Israel has three primary climate zones: (1) a Mediterranean region in the central and northern areas, (2) an arid desert zone in the southern and eastern parts, and (3) a semi-arid transition zone situated between the Mediterranean and desert areas [44]. The prevalence of *Leishmania* infections typically corresponds with these climate zones. Whereas *L. infantum* infections have been predominantly reported in the Mediterranean region, *L. major* and *L. tropica* infections have been mostly documented in arid and semi-arid regions, while *L. donovani* infections were described only in arid zones [12,19]. The distribution of sand fly species identified in this study corresponds with these infection patterns and supports prior findings about the geographic prevalence of human and animal *Leishmania* infections. The principal vectors of *L. major* and *L. donovani* were primarily found in southern Israel, while the vectors of *L. tropica* and *L. infantum* were more commonly detected in central and northern Israel.

Unexpectedly, certain sand fly vectors were identified beyond their historically recognized regions. *Phlebotomus sergenti*, the principal vector of *L. tropica*, was identified in southern Israel, whereas *Ph. alexandri*, a recognized vector of *L. donovani*, was observed in middle and northern Israel. These findings suggest that sand fly vector distribution in Israel is not strictly confined to expected areas, and that *Leishmania* transmission could occur beyond traditionally recognized endemic regions. These shifts in distribution underscore the necessity for ongoing surveillance and further studies to determine if these changes are driven by climate change, anthropogenic activities, or ecological shifts, as these factors may contribute to the expansion of sand fly habitats and the transmission of *Leishmania* to humans and animals.

The detection of 25 different mammalian blood meal sources, including both domestic and wild animals, highlights the complex epidemiology of the leishmaniasis transmission cycle in Israel and the Middle East. The majority of the blood meals derived from just four animal hosts: domestic cats, rock hyraxes, European hares, and cows. This is unexpected, as these animal species are not necessarily the most abundant or largest in terms of body surface area available for sand fly feeding. The composition of blood meals is likely influenced by sand fly host-feeding preferences, which can vary between sand fly species and might be shaped by environmental factors [45,46]. Identifying of blood meal sources and understanding their relationship to *Leishmania* transmission are essential components devising effective prevention strategies to reduce the spread of leishmaniasis [19,47–49].

In conclusion, the HRM-PCR methods developed in this study enable the simultaneous detection of sand fly species, characterization of blood meals, and identification of *Leishmania* infection and parasite species discrimination. These methods have provided valuable insights into the distribution of sand fly species in Israel, their predominant blood meal sources, and their infection status with various *Leishmania* species in Israel. These techniques can be applied in entomological and epidemiological studies, as well as in clinical settings for the rapid diagnosis of *Leishmania* species, supporting treatment decisions and prognosis. Ultimately, this method holds significant potential to enhance leishmaniasis surveillance and contribute to disease control efforts.

## Supporting information

S1 Fig Human *Leishmania* morbidity cases number by year (2000–2023) in Israel. Data reported by the Department of Epidemiology, Ministry of Health of Israel. VL – visceral leishmaniasis, CL – cutaneous leishmaniasis.
(TIF)

S2 Fig Distribution of phlebotomine sand fly species which are not vectors of *Leishmania* species identified in this study. Map was created using ArcGIS software. Base map source: Natural Earth (https://www.naturalearthdata.com/), public domain.
(TIF)

S1 Table Complete data of sand fly samples identification by morphology, HRM-RT-PCR for the *cytochrome b* (*cytb*) gene, and sequencing. Species with different morphology identification are marked by red.
(DOCX)

S2 Table Species of engorged sand flies and their blood meal sources detected in the study. (DOCX)

S3 Table Reference amplicon melting temperatures (Tm) obtained by high-resolution melting (HRM) analysis for the identification of *Leishmania* species, sand fly vectors, and vertebrate blood meal sources. (DOCX)

S1 File Multiple sequence alignments for HRM Targets. (DOCX)
