# Supplementary figures and images for: A multifaceted molecular approach to surveillance of leishmaniasis: Identification of sand fly species, Leishmania parasites, and blood meal sources using high-resolution melting analysis
Source: PLoS Negl Trop Dis. 2025 Sep 24;19(9):e0013412. doi: 10.1371/journal.pntd.0013412 (PMC12503242; doi:10.1371/journal.pntd.0013412)

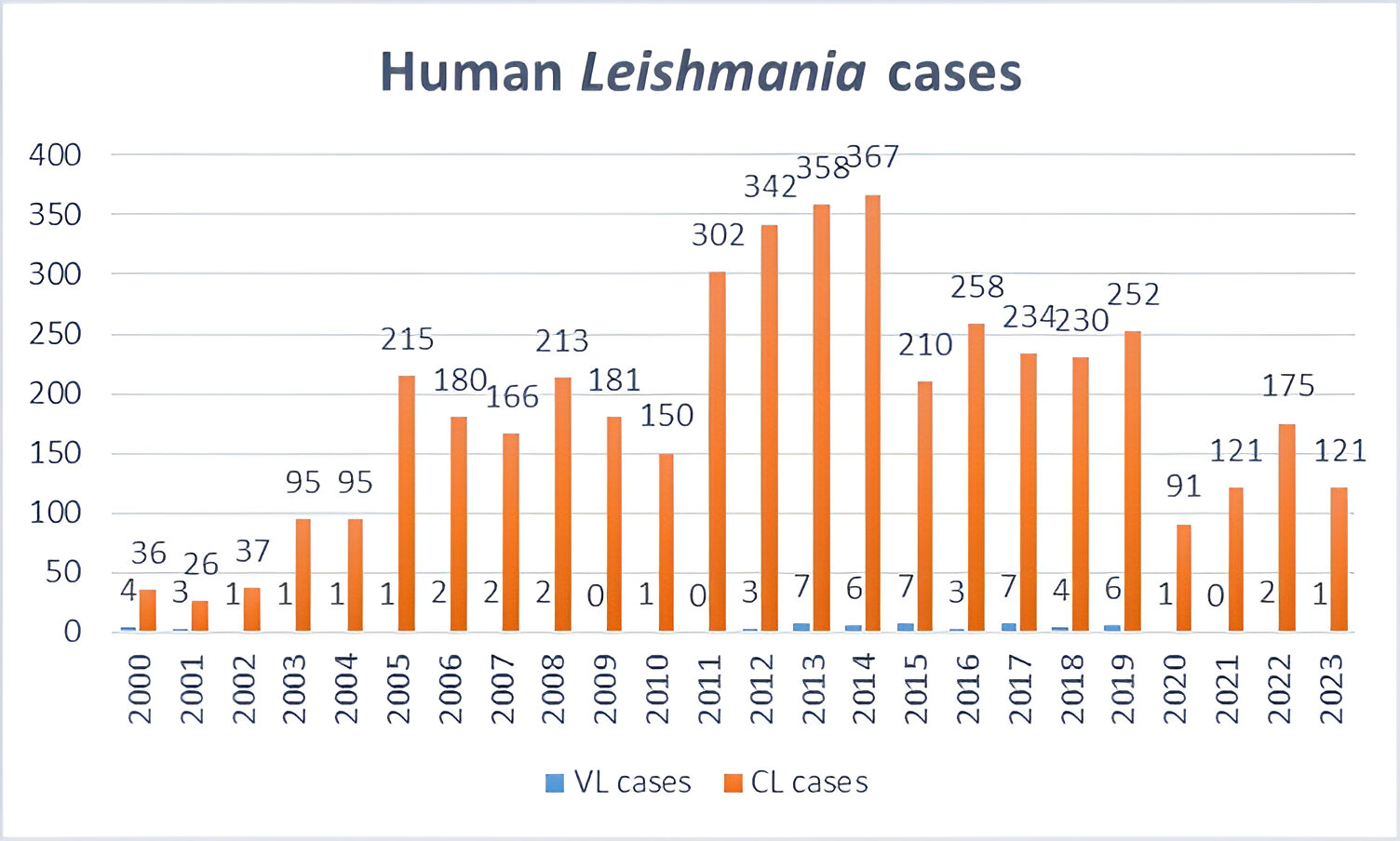

Supplement: S1 Fig — Data reported by the Department of Epidemiology, Ministry of Health of Israel. VL – visceral leishmaniasis, CL – cutaneous leishmaniasis. (TIF) [file pntd.0013412.s001.tif]

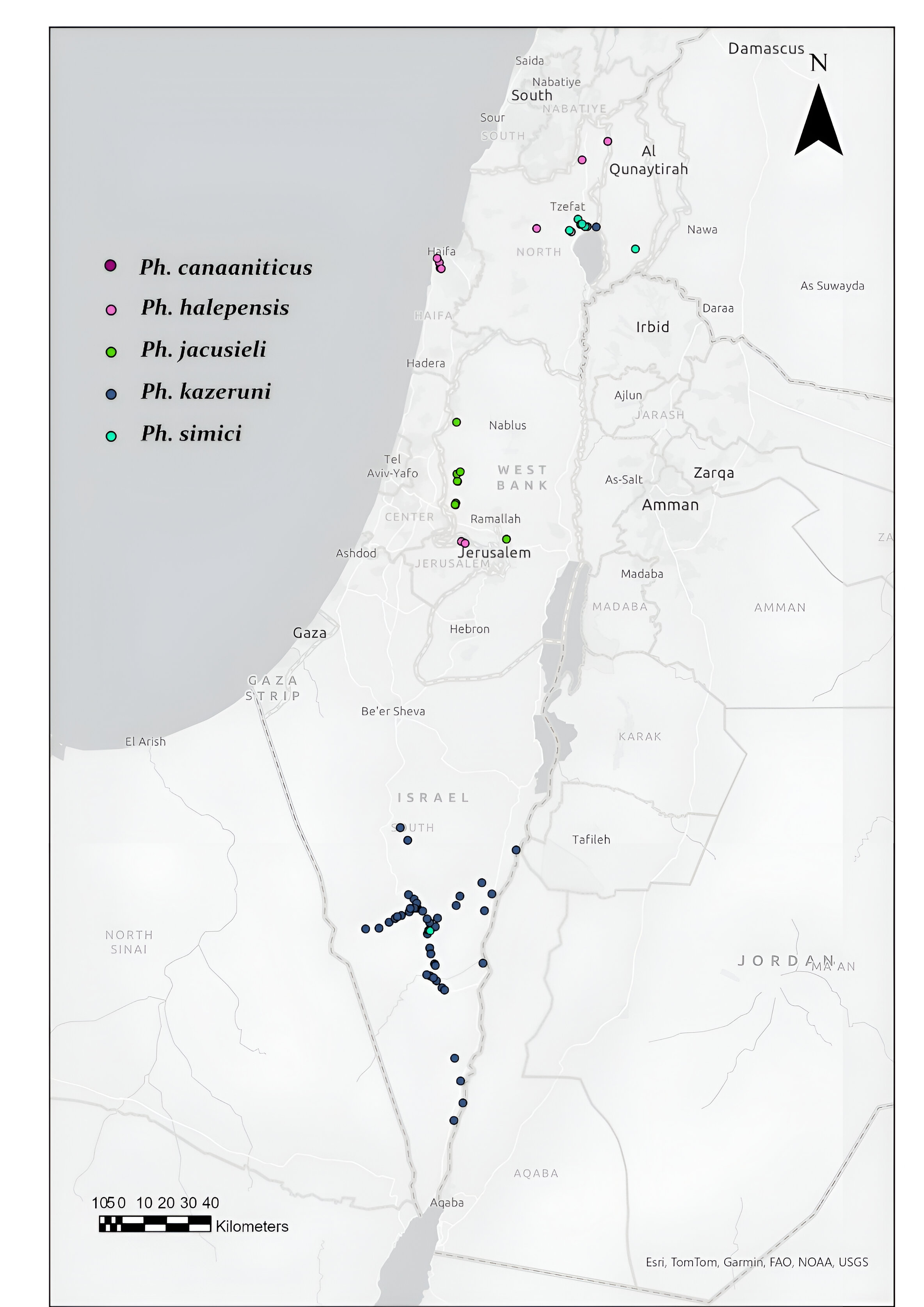

Supplement: S2 Fig — Map was created using ArcGIS software. Base map source: Natural Earth (https://www.naturalearthdata.com/), public domain. (TIF) [file pntd.0013412.s002.tif]
