# Supplementary material for: A multifaceted molecular approach to surveillance of leishmaniasis: Identification of sand fly species, Leishmania parasites, and blood meal sources using high-resolution melting analysis
Source: PLoS Negl Trop Dis. 2025 Sep 24;19(9):e0013412. doi: 10.1371/journal.pntd.0013412 (PMC12503242; doi:10.1371/journal.pntd.0013412)
Supplement: S1 Table — Species with different morphology identification are marked by red. (DOCX) [file pntd.0013412.s003.docx]

| **no.** | **Sample name** | **Sand fly species** | **Sex** | **Morphology identification** | **HRM RT-PCR ID** | **SEQ ID** | **GenBank accession number used for species identification** |
| --- | --- | --- | --- | --- | --- | --- | --- |
| **1** | Palex1 | *Ph. alexandri* | Female | *Ph. alexandri* | *Ph. alexandri* | *Ph. alexandri* | PQ868583 |
| **2** | Palex2 | *Ph. alexandri* | Female | *Ph. alexandri* | *Ph. alexandri* | *Ph. alexandri* |  |
| **3** | Palex3 | *Ph. alexandri* | Female | *Ph. alexandri* | *Ph. alexandri* | *Ph. alexandri* |  |
| **4** | Palex4 | *Ph. alexandri* | Female | *Ph. alexandri* | *Ph. alexandri* | *Ph. alexandri* |  |
| **5** | Palex5 | *Ph. alexandri* | Female | *Ph. alexandri* | *Ph. alexandri* | *Ph. alexandri* |  |
| **6** | Palex6 | *Ph. alexandri* | Female | *Ph. alexandri* | *Ph. alexandri* | *Ph. alexandri* |  |
| **7** | Palex7 | *Ph. alexandri* | Female | *Ph. alexandri* | *Ph. alexandri* | *Ph. alexandri* |  |
| **8** | Palex8 | *Ph. alexandri* | Female | *Ph. alexandri* | *Ph. alexandri* | *Ph. alexandri* |  |
| **9** | Palex10 | *Ph. alexandri* | Female | *Ph. alexandri* | *Ph. alexandri* | *Ph. alexandri* |  |
| **10** | Palex11 | *Ph. alexandri* | Female | *Ph. alexandri* | *Ph. alexandri* | *Ph. alexandri* | PQ868584 |
| **11** | Palex12 | *Ph. alexandri* | Female | *Ph. alexandri* | *Ph. alexandri* | *Ph. alexandri* | PQ868583 |
| **12** | Palex13 | *Ph. alexandri* | Female | *Ph. alexandri* | *Ph. alexandri* | *Ph. alexandri* |  |
| **13** | Palex14 | *Ph. alexandri* | Male | *Ph. alexandri* | *Ph. alexandri* | *Ph. alexandri* |  |
| **14** | Palex15 | *Ph. alexandri* | Male | *Ph. alexandri* | *Ph. alexandri* | *Ph. alexandri* | PQ868584 |
| **15** | Palex16 | *Ph. alexandri* | Male | *Ph. alexandri* | *Ph. alexandri* | *Ph. alexandri* | PQ868583 |
| **16** | Palex17 | *Ph. alexandri* | Male | *Ph. alexandri* | *Ph. alexandri* | *Ph. alexandri* |  |
| **17** | Palex18 | *Ph. alexandri* | Male | *Ph. alexandri* | *Ph. alexandri* | *Ph. alexandri* |  |
| **18** | Palex19 | *Ph. alexandri* | Male | *Ph. alexandri* | *Ph. alexandri* | *Ph. alexandri* |  |
| **19** | Palex20 | *Ph. alexandri* | Male | *Ph. alexandri* | *Ph. alexandri* | *Ph. alexandri* |  |
| **20** | Palex21 | *Ph. alexandri* | Male | *Ph. alexandri* | *Ph. alexandri* | *Ph. alexandri* |  |
| **21** | Palex22 | *Ph. alexandri* | Male | *Ph. alexandri* | *Ph. alexandri* | *Ph. alexandri* |  |
| **22** | Palex23 | *Ph. alexandri* | Male | *Ph. alexandri* | *Ph. alexandri* | *Ph. alexandri* |  |
| **23** | Palex24 | *Ph. alexandri* | Male | *Ph. alexandri* | *Ph. alexandri* | *Ph. alexandri* |  |
| **24** | Palex25 | *Ph. alexandri* | Male | *Ph. alexandri* | *Ph. alexandri* | *Ph. alexandri* | PQ868584 |
| **25** | Palex26 | *Ph. alexandri* | Male | *Ph. alexandri* | *Ph. alexandri* | *Ph. alexandri* | PQ868583 |
| **26** | Palex27 | *Ph. alexandri* | Male | *Ph. alexandri* | *Ph. alexandri* | *Ph. alexandri* |  |
| **27** | Palex28 | *Ph. alexandri* | Male | *Ph. alexandri* | *Ph. alexandri* | *Ph. alexandri* | PQ868584 |
| **28** | Parab1 | *Ph. arabicus* | Female | *Ph. arabicus* | *Ph. arabicus* | *Ph. arabicus* | PQ852116 |
| **29** | Parab2 | *Ph. arabicus* | Female | *Ph. arabicus* | *Ph. arabicus* | *Ph. arabicus* |  |
| **30** | Parab3 | *Ph. arabicus* | Female | *Ph. arabicus* | *Ph. arabicus* | *Ph. arabicus* |  |
| **31** | Parab4 | *Ph. arabicus* | Female | *Ph. arabicus* | *Ph. arabicus* | *Ph. arabicus* |  |
| **32** | Parab5 | *Ph. arabicus* | Female | *Ph. arabicus* | *Ph. arabicus* | *Ph. arabicus* |  |
| **33** | Parab6 | *Ph. arabicus* | Female | *Ph. arabicus* | *Ph. arabicus* | *Ph. arabicus* |  |
| **34** | Parab13 | *Ph. arabicus* | Male | *Ph. arabicus* | *Ph. arabicus* | *Ph. arabicus* | PQ852117 |
| **35** | Parab14 | *Ph. arabicus* | Male | *Ph. arabicus* | *Ph. arabicus* | *Ph. arabicus* | PQ852116 |
| **36** | Parab15 | *Ph. arabicus* | Male | *Ph. arabicus* | *Ph. arabicus* | *Ph. arabicus* | PQ852117 |
| **37** | Parab16 | *Ph. arabicus* | Male | *Ph. arabicus* | *Ph. arabicus* | *Ph. arabicus* |  |
| **38** | Parab17 | *Ph. arabicus* | Male | *Ph. arabicus* | *Ph. arabicus* | *Ph. arabicus* |  |
| **39** | Parab18 | *Ph. arabicus* | Male | *Ph. arabicus* | *Ph. arabicus* | *Ph. arabicus* | PQ852116 |
| **40** | Parab19 | *Ph. arabicus* | Male | *Ph. simici* | *Ph. arabicus* | *Ph. arabicus* |  |
| **41** | Parab20 | *Ph. arabicus* | Male | *Ph. simici* | *Ph. arabicus* | *Ph. arabicus* | PQ852117 |
| **42** | Parab21 | *Ph. arabicus* | Male | *Ph. halepensis* | *Ph. arabicus* | *Ph. arabicus* |  |
| **43** | Parab22 | *Ph. arabicus* | Male | *Ph. arabicus* | *Ph. arabicus* | *Ph. arabicus* | PQ852116 |
| **44** | Parab23 | *Ph. arabicus* | Male | *Ph. arabicus* | *Ph. arabicus* | *Ph. arabicus* |  |
| **45** | Pcan1 | *Ph. canaaniticus* | Female | *Ph. canaaniticus* | *Ph. canaaniticus* | *Ph. canaaniticus* | PQ815623 |
| **46** | Pcan2 | *Ph. canaaniticus* | Female | *Ph. canaaniticus* | *Ph. canaaniticus* | *Ph. canaaniticus* |  |
| **47** | Pcan3 | *Ph. canaaniticus* | Female | *Ph. canaaniticus* | *Ph. canaaniticus* | *Ph. canaaniticus* |  |
| **48** | Pcan4 | *Ph. canaaniticus* | Female | *Ph. canaaniticus* | *Ph. canaaniticus* | *Ph. canaaniticus* |  |
| **49** | Pcan5 | *Ph. canaaniticus* | Female | *Ph. canaaniticus* | *Ph. canaaniticus* | *Ph. canaaniticus* |  |
| **50** | Pcan6 | *Ph. canaaniticus* | Male | *Ph. canaaniticus* | *Ph. canaaniticus* | *Ph. canaaniticus* |  |
| **51** | Pcan7 | *Ph. canaaniticus* | Male | *Ph. canaaniticus* | *Ph. canaaniticus* | *Ph. canaaniticus* |  |
| **52** | Pcan8 | *Ph. canaaniticus* | Male | *Ph. canaaniticus* | *Ph. canaaniticus* | *Ph. canaaniticus* |  |
| **53** | Pcan9 | *Ph. canaaniticus* | Male | *Ph. canaaniticus* | *Ph. canaaniticus* | *Ph. canaaniticus* |  |
| **54** | Phal1 | *Ph. halepensis* | Female | *Ph. halepensis* | *Ph. halepensis* | *Ph. halepensis* | PQ852115 |
| **55** | Phal2 | *Ph. halepensis* | Female | *Ph. halepensis* | *Ph. halepensis* | *Ph. halepensis* |  |
| **56** | Phal3 | *Ph. halepensis* | Female | *Ph. halepensis* | *Ph. halepensis* | *Ph. halepensis* |  |
| **57** | Phal4 | *Ph. halepensis* | Female | *Ph. halepensis* | *Ph. halepensis* | *Ph. halepensis* |  |
| **58** | Phal5 | *Ph. halepensis* | Female | *Ph. halepensis* | *Ph. halepensis* | *Ph. halepensis* |  |
| **59** | Phal6 | *Ph. halepensis* | Female | *Ph. halepensis* | *Ph. halepensis* | *Ph. halepensis* |  |
| **60** | Phal7 | *Ph. halepensis* | Female | *Ph. halepensis* | *Ph. halepensis* | *Ph. halepensis* |  |
| **61** | Phal9 | *Ph. halepensis* | Female | *Ph. halepensis* | *Ph. halepensis* | *Ph. halepensis* |  |
| **62** | Phal10 | *Ph. halepensis* | Female | *Ph. halepensis* | *Ph. halepensis* | *Ph. halepensis* |  |
| **63** | Phal11 | *Ph. halepensis* | Female | *Ph. halepensis* | *Ph. halepensis* | *Ph. halepensis* |  |
| **64** | Phal12 | *Ph. halepensis* | Female | *Ph. sergenti* | *Ph. halepensis* | *Ph. halepensis* |  |
| **65** | Phal13 | *Ph. halepensis* | Male | *Ph. halepensis* | *Ph. halepensis* | *Ph. halepensis* |  |
| **66** | Phal14 | *Ph. halepensis* | Male | *Ph. halepensis* | *Ph. halepensis* | *Ph. halepensis* |  |
| **67** | Phal16 | *Ph. halepensis* | Male | *Ph. halepensis* | *Ph. halepensis* | *Ph. halepensis* |  |
| **68** | Phal17 | *Ph. halepensis* | Male | *Ph. halepensis* | *Ph. halepensis* | *Ph. halepensis* |  |
| **69** | Phal18 | *Ph. halepensis* | Male | *Ph. halepensis* | *Ph. halepensis* | *Ph. halepensis* |  |
| **70** | Phal19 | *Ph. halepensis* | Male | *Ph. halepensis* | *Ph. halepensis* | *Ph. halepensis* |  |
| **71** | Pjac1 | *Ph. jacusieli* | Female | *Ph. jacusieli* | *Ph. jacusieli* | *Ph. jacusieli* | PQ868585 |
| **72** | Pjac2 | *Ph. jacusieli* | Female | *Ph. jacusieli* | *Ph. jacusieli* | *Ph. jacusieli* |  |
| **73** | Pjac3 | *Ph. jacusieli* | Female | *Ph. jacusieli* | *Ph. jacusieli* | *Ph. jacusieli* |  |
| **74** | Pjac4 | *Ph. jacusieli* | Female | *Ph. jacusieli* | *Ph. jacusieli* | *Ph. jacusieli* |  |
| **75** | Pjac5 | *Ph. jacusieli* | Female | *Ph. jacusieli* | *Ph. jacusieli* | *Ph. jacusieli* |  |
| **76** | Pjac12 | *Ph. jacusieli* | Male | *Ph. jacusieli* | *Ph. jacusieli* | *Ph. jacusieli* |  |
| **77** | Pjac13 | *Ph. jacusieli* | Male | *Paraphlebotomus sp.* | *Ph. jacusieli* | *Ph. jacusieli* |  |
| **78** | Pjac14 | *Ph. jacusieli* | Male | *Ph. jacusieli* | *Ph. jacusieli* | *Ph. jacusieli* |  |
| **79** | Pjac15 | *Ph. jacusieli* | Male | *Ph. jacusieli* | *Ph. jacusieli* | *Ph. jacusieli* |  |
| **80** | Pjac16 | *Ph. jacusieli* | Male | *Ph. jacusieli* | *Ph. jacusieli* | *Ph. jacusieli* |  |
| **81** | Pjac17 | *Ph. jacusieli* | Male | *Ph. jacusieli* | *Ph. jacusieli* | *Ph. jacusieli* |  |
| **82** | Pkaz1 | *Ph. kazeruni* | Female | *Ph. kazeruni* | *Ph. kazeruni* | *Ph. kazeruni* | PQ852118 |
| **83** | Pkaz2 | *Ph. kazeruni* | Female | *Ph. kazeruni* | *Ph. kazeruni* | *Ph. kazeruni* |  |
| **84** | Pkaz3 | *Ph. kazeruni* | Female | *Ph. kazeruni* | *Ph. kazeruni* | *Ph. kazeruni* | PQ868586 |
| **85** | Pkaz5 | *Ph. kazeruni* | Female | *Ph. kazeruni* | *Ph. kazeruni* | *Ph. kazeruni* | PQ852118 |
| **86** | Pkaz6 | *Ph. kazeruni* | Female | *Ph. kazeruni* | *Ph. kazeruni* | *Ph. kazeruni* |  |
| **87** | Pkaz7 | *Ph. kazeruni* | Female | *Ph. kazeruni* | *Ph. kazeruni* | *Ph. kazeruni* |  |
| **88** | Pkaz8 | *Ph. kazeruni* | Female | *Ph. kazeruni* | *Ph. kazeruni* | *Ph. kazeruni* |  |
| **89** | Pkaz9 | *Ph. kazeruni* | Female | *Ph. kazeruni* | *Ph. kazeruni* | *Ph. kazeruni* |  |
| **90** | Pkaz10 | *Ph. kazeruni* | Female | *Ph. kazeruni* | *Ph. kazeruni* | *Ph. kazeruni* |  |
| **91** | Pkaz11 | *Ph. kazeruni* | Female | *Ph. kazeruni* | *Ph. kazeruni* | *Ph. kazeruni* |  |
| **92** | Pkaz12 | *Ph. kazeruni* | Female | *Ph. kazeruni* | *Ph. kazeruni* | *Ph. kazeruni* |  |
| **93** | Pkaz13 | *Ph. kazeruni* | Female | *Ph. kazeruni* | *Ph. kazeruni* | *Ph. kazeruni* |  |
| **94** | Pkaz14 | *Ph. kazeruni* | Female | *Ph. kazeruni* | *Ph. kazeruni* | *Ph. kazeruni* | PQ868586 |
| **95** | Pkaz15 | *Ph. kazeruni* | Female | *Ph. kazeruni* | *Ph. kazeruni* | *Ph. kazeruni* | PQ852118 |
| **96** | Pkaz16 | *Ph. kazeruni* | Male | *Ph. kazeruni* | *Ph. kazeruni* | *Ph. kazeruni* |  |
| **97** | Pkaz17 | *Ph. kazeruni* | Male | *Ph. kazeruni* | *Ph. kazeruni* | *Ph. kazeruni* |  |
| **98** | Pkaz18 | *Ph. kazeruni* | Male | *Ph. kazeruni* | *Ph. kazeruni* | *Ph. kazeruni* |  |
| **99** | Pkaz19 | *Ph. kazeruni* | Male | *Ph. kazeruni* | *Ph. kazeruni* | *Ph. kazeruni* | PQ852119 |
| **100** | Pkaz20 | *Ph. kazeruni* | Male | *Ph. kazeruni* | *Ph. kazeruni* | *Ph. kazeruni* | PQ852118 |
| **101** | Pkaz21 | *Ph. kazeruni* | Male | *Ph. kazeruni* | *Ph. kazeruni* | *Ph. kazeruni* | PQ852119 |
| **102** | Pkaz22 | *Ph. kazeruni* | Male | *Ph. kazeruni* | *Ph. kazeruni* | *Ph. kazeruni* | PQ852118 |
| **103** | Pkaz23 | *Ph. kazeruni* | Male | *Ph. kazeruni* | *Ph. kazeruni* | *Ph. kazeruni* | PQ852119 |
| **104** | Pkaz24 | *Ph. kazeruni* | Male | *Ph. kazeruni* | *Ph. kazeruni* | *Ph. kazeruni* | PQ868586 |
| **105** | Pkaz25 | *Ph. kazeruni* | Male | *Ph. kazeruni* | *Ph. kazeruni* | *Ph. kazeruni* | PQ852119 |
| **106** | Pkaz26 | *Ph. kazeruni* | Male | *Ph. kazeruni* | *Ph. kazeruni* | *Ph. kazeruni* | PQ868586 |
| **107** | Pkaz27 | *Ph. kazeruni* | Male | *Ph. kazeruni* | *Ph. kazeruni* | *Ph. kazeruni* | PQ852118 |
| **108** | Pkaz28 | *Ph. kazeruni* | Male | *Ph. kazeruni* | *Ph. kazeruni* | *Ph. kazeruni* | PQ868586 |
| **109** | Pkaz29 | *Ph. kazeruni* | Male | *Ph. kazeruni* | *Ph. kazeruni* | *Ph. kazeruni* |  |
| **110** | Pkaz30 | *Ph. kazeruni* | Male | *Ph. kazeruni* | *Ph. kazeruni* | *Ph. kazeruni* |  |
| **111** | Ppap1 | *Ph. papatasi* | Female | *Ph. papatasi* | *Ph. papatasi* | *Ph. papatasi* | PQ868587 |
| **112** | Ppap2 | *Ph. papatasi* | Female | *Ph. papatasi* | *Ph. papatasi* | *Ph. papatasi* |  |
| **113** | Ppap4 | *Ph. papatasi* | Female | *Ph. papatasi* | *Ph. papatasi* | *Ph. papatasi* |  |
| **114** | Ppap5 | *Ph. papatasi* | Female | *Ph. papatasi* | *Ph. papatasi* | *Ph. papatasi* | PQ852120 |
| **115** | Ppap6 | *Ph. papatasi* | Female | *Ph. papatasi* | *Ph. papatasi* | *Ph. papatasi* | PQ868587 |
| **116** | Ppap7 | *Ph. papatasi* | Female | *Ph. papatasi* | *Ph. papatasi* | *Ph. papatasi* | PQ852121 |
| **117** | Ppap8 | *Ph. papatasi* | Female | *Ph. papatasi* | *Ph. papatasi* | *Ph. papatasi* |  |
| **118** | Ppap9 | *Ph. papatasi* | Female | *Ph. papatasi* | *Ph. papatasi* | *Ph. papatasi* | PQ852120 |
| **119** | Ppap10 | *Ph. papatasi* | Female | *Ph. papatasi* | *Ph. papatasi* | *Ph. papatasi* | PQ852121 |
| **120** | Ppap11 | *Ph. papatasi* | Female | *Ph. papatasi* | *Ph. papatasi* | *Ph. papatasi* | PQ852120 |
| **121** | Ppap12 | *Ph. papatasi* | Female | *Ph. papatasi* | *Ph. papatasi* | *Ph. papatasi* |  |
| **122** | Ppap13 | *Ph. papatasi* | Female | *Ph. papatasi* | *Ph. papatasi* | *Ph. papatasi* | PQ852121 |
| **123** | Ppap14 | *Ph. papatasi* | Female | *Ph. papatasi* | *Ph. papatasi* | *Ph. papatasi* | PQ852120 |
| **124** | Ppap15 | *Ph. papatasi* | Female | *Ph. papatasi* | *Ph. papatasi* | *Ph. papatasi* |  |
| **125** | Ppap16 | *Ph. papatasi* | Female | *Ph. papatasi* | *Ph. papatasi* | *Ph. papatasi* | PQ852121 |
| **126** | Ppap17 | *Ph. papatasi* | Female | *Ph. papatasi* | *Ph. papatasi* | *Ph. papatasi* | PQ852120 |
| **127** | Ppap18 | *Ph. papatasi* | Male | *Ph. papatasi* | *Ph. papatasi* | *Ph. papatasi* | PQ852121 |
| **128** | Ppap19 | *Ph. papatasi* | Male | *Ph. papatasi* | *Ph. papatasi* | *Ph. papatasi* |  |
| **129** | Ppap20 | *Ph. papatasi* | Male | *Ph. papatasi* | *Ph. papatasi* | *Ph. papatasi* |  |
| **130** | Ppap21 | *Ph. papatasi* | Male | *Ph. papatasi* | *Ph. papatasi* | *Ph. papatasi* |  |
| **131** | Ppap22 | *Ph. papatasi* | Male | *Ph. papatasi* | *Ph. papatasi* | *Ph. papatasi* | PQ852120 |
| **132** | Ppap23 | *Ph. papatasi* | Male | *Ph. papatasi* | *Ph. papatasi* | *Ph. papatasi* | PQ868587 |
| **133** | Ppap24 | *Ph. papatasi* | Male | *Ph. papatasi* | *Ph. papatasi* | *Ph. papatasi* |  |
| **134** | Ppap25 | *Ph. papatasi* | Male | *Ph. papatasi* | *Ph. papatasi* | *Ph. papatasi* |  |
| **135** | Ppap26 | *Ph. papatasi* | Male | *Ph. papatasi* | *Ph. papatasi* | *Ph. papatasi* | PQ852120 |
| **136** | Ppap27 | *Ph. papatasi* | Male | *Ph. papatasi* | *Ph. papatasi* | *Ph. papatasi* | PQ868587 |
| **137** | Ppap28 | *Ph. papatasi* | Male | *Ph. papatasi* | *Ph. papatasi* | *Ph. papatasi* | PQ852121 |
| **138** | Ppap29 | *Ph. papatasi* | Male | *Ph. papatasi* | *Ph. papatasi* | *Ph. papatasi* |  |
| **139** | Ppap30 | *Ph. papatasi* | Male | *Ph. papatasi* | *Ph. papatasi* | *Ph. papatasi* |  |
| **140** | Pper1 | *Ph. perfiliewi galilaeus* | Female | *Ph. perfiliewi galilaeus* | *Ph. perfiliewi galilaeus* | *Ph. perfiliewi galilaeus* | PQ852131 |
| **141** | Pper2 | *Ph. perfiliewi galilaeus* | Female | *Ph. perfiliewi galilaeus* | *Ph. perfiliewi galilaeus* | *Ph. perfiliewi galilaeus* | PQ852128 |
| **142** | Pper3 | *Ph. perfiliewi galilaeus* | Female | *Ph. perfiliewi galilaeus* | *Ph. perfiliewi galilaeus* | *Ph. perfiliewi galilaeus* | PQ852132 |
| **143** | Pper4 | *Ph. perfiliewi galilaeus* | Female | *Ph. perfiliewi galilaeus* | *Ph. perfiliewi galilaeus* | *Ph. perfiliewi galilaeus* | PQ852135 |
| **144** | Pper5 | *Ph. perfiliewi galilaeus* | Female | *Ph. perfiliewi galilaeus* | *Ph. perfiliewi galilaeus* | *Ph. perfiliewi galilaeus* | PQ852120 |
| **145** | Pper6 | *Ph. perfiliewi galilaeus* | Female | *Ph. perfiliewi galilaeus* | *Ph. perfiliewi galilaeus* | *Ph. perfiliewi galilaeus* | PQ852131 |
| **146** | Pper7 | *Ph. perfiliewi galilaeus* | Female | *Ph. perfiliewi galilaeus* | *Ph. perfiliewi galilaeus* | *Ph. perfiliewi galilaeus* | PQ852126 |
| **147** | Pper8 | *Ph. perfiliewi galilaeus* | Female | *Ph. perfiliewi galilaeus* | *Ph. perfiliewi galilaeus* | *Ph. perfiliewi galilaeus* | PQ852134 |
| **148** | Pper9 | *Ph. perfiliewi galilaeus* | Female | *Ph. perfiliewi galilaeus* | *Ph. perfiliewi galilaeus* | *Ph. perfiliewi galilaeus* | PQ852135 |
| **149** | Pper10 | *Ph. perfiliewi galilaeus* | Female | *Ph. perfiliewi galilaeus* | *Ph. perfiliewi galilaeus* | *Ph. perfiliewi galilaeus* | PQ852128 |
| **150** | Pper11 | *Ph. perfiliewi galilaeus* | Female | *Ph. perfiliewi galilaeus* | *Ph. perfiliewi galilaeus* | *Ph. perfiliewi galilaeus* |  |
| **151** | Pper12 | *Ph. perfiliewi galilaeus* | Female | *Ph. perfiliewi galilaeus* | *Ph. perfiliewi galilaeus* | *Ph. perfiliewi galilaeus* | PQ852126 |
| **152** | Pper13 | *Ph. perfiliewi galilaeus* | Female | *Ph. perfiliewi galilaeus* | *Ph. perfiliewi galilaeus* | *Ph. perfiliewi galilaeus* | PQ852130 |
| **153** | Pper14 | *Ph. perfiliewi galilaeus* | Female | *Ph. perfiliewi galilaeus* | *Ph. perfiliewi galilaeus* | *Ph. perfiliewi galilaeus* | PQ852133 |
| **154** | Pper15 | *Ph. perfiliewi galilaeus* | Female | *Ph. perfiliewi galilaeus* | *Ph. perfiliewi galilaeus* | *Ph. perfiliewi galilaeus* | PQ852127 |
| **155** | Pper16 | *Ph. perfiliewi galilaeus* | Male | *Ph. perfiliewi galilaeus* | *Ph. perfiliewi galilaeus* | *Ph. perfiliewi galilaeus* | PQ852128 |
| **156** | Pper17 | *Ph. perfiliewi galilaeus* | Male | *Ph. perfiliewi galilaeus* | *Ph. perfiliewi galilaeus* | *Ph. perfiliewi galilaeus* | PQ852131 |
| **157** | Pper20 | *Ph. perfiliewi galilaeus* | Male | *Ph. perfiliewi galilaeus* | *Ph. perfiliewi galilaeus* | *Ph. perfiliewi galilaeus* | PQ852128 |
| **158** | Pper21 | *Ph. perfiliewi galilaeus* | Male | *Ph. perfiliewi galilaeus* | *Ph. perfiliewi galilaeus* | *Ph. perfiliewi galilaeus* | PQ852131 |
| **159** | Pper22 | *Ph. perfiliewi galilaeus* | Male | *Ph. perfiliewi galilaeus* | *Ph. perfiliewi galilaeus* | *Ph. perfiliewi galilaeus* | PQ852135 |
| **160** | Pper23 | *Ph. perfiliewi galilaeus* | Male | *Ph. perfiliewi galilaeus* | *Ph. perfiliewi galilaeus* | *Ph. perfiliewi galilaeus* |  |
| **161** | Pper24 | *Ph. perfiliewi galilaeus* | Male | *Ph. perfiliewi galilaeus* | *Ph. perfiliewi galilaeus* | *Ph. perfiliewi galilaeus* |  |
| **162** | Pper25 | *Ph. perfiliewi galilaeus* | Male | *Ph. perfiliewi galilaeus* | *Ph. perfiliewi galilaeus* | *Ph. perfiliewi galilaeus* | PQ852126 |
| **163** | Pper26 | *Ph. perfiliewi galilaeus* | Male | *Ph. perfiliewi galilaeus* | *Ph. perfiliewi galilaeus* | *Ph. perfiliewi galilaeus* | PQ852131 |
| **164** | Pper27 | *Ph. perfiliewi galilaeus* | Male | *Ph. perfiliewi galilaeus* | *Ph. perfiliewi galilaeus* | *Ph. perfiliewi galilaeus* | PQ852135 |
| **165** | Pper28 | *Ph. perfiliewi galilaeus* | Male | *Ph. syriacus* | *Ph. perfiliewi galilaeus* | *Ph. perfiliewi galilaeus* | PQ852133 |
| **166** | Pser3 | *Ph. sergenti* | Female | *Ph. sergenti* | *Ph. sergenti* | *Ph. sergenti* | PQ868589 |
| **167** | Pser4 | *Ph. sergenti* | Female | *Ph. sergenti* | *Ph. sergenti* | *Ph. sergenti* |  |
| **168** | Pser5 | *Ph. sergenti* | Female | *Ph. sergenti* | *Ph. sergenti* | *Ph. sergenti* |  |
| **169** | Pser6 | *Ph. sergenti* | Female | *Ph. sergenti* | *Ph. sergenti* | *Ph. sergenti* |  |
| **170** | Pser7 | *Ph. sergenti* | Female | *Ph. sergenti* | *Ph. sergenti* | *Ph. sergenti* |  |
| **171** | Pser8 | *Ph. sergenti* | Female | *Ph. sergenti* | *Ph. sergenti* | *Ph. sergenti* |  |
| **172** | Pser9 | *Ph. sergenti* | Female | *Ph. sergenti* | *Ph. sergenti* | *Ph. sergenti* |  |
| **173** | Pser10 | *Ph. sergenti* | Female | *Ph. sergenti* | *Ph. sergenti* | *Ph. sergenti* |  |
| **174** | Pser11 | *Ph. sergenti* | Female | *Ph. sergenti* | *Ph. sergenti* | *Ph. sergenti* | PQ852122 |
| **175** | Pser12 | *Ph. sergenti* | Female | *Ph. sergenti* | *Ph. sergenti* | *Ph. sergenti* |  |
| **176** | Pser13 | *Ph. sergenti* | Female | *Ph. sergenti* | *Ph. sergenti* | *Ph. sergenti* | PQ868589 |
| **177** | Pser14 | *Ph. sergenti* | Female | *Ph. sergenti* | *Ph. sergenti* | *Ph. sergenti* |  |
| **178** | Pser15 | *Ph. sergenti* | Female | *Ph. sergenti* | *Ph. sergenti* | *Ph. sergenti* |  |
| **179** | Pser16 | *Ph. sergenti* | Female | *Ph. sergenti* | *Ph. sergenti* | *Ph. sergenti* | PQ852122 |
| **180** | Pser18 | *Ph. sergenti* | Male | *Ph. sergenti* | *Ph. sergenti* | *Ph. sergenti* | PQ868589 |
| **181** | Pser19 | *Ph. sergenti* | Male | *Ph. sergenti* | *Ph. sergenti* | *Ph. sergenti* |  |
| **182** | Pser20 | *Ph. sergenti* | Male | *Ph. sergenti* | *Ph. sergenti* | *Ph. sergenti* |  |
| **183** | Pser21 | *Ph. sergenti* | Male | *Ph. sergenti* | *Ph. sergenti* | *Ph. sergenti* |  |
| **184** | Pser22 | *Ph. sergenti* | Male | *Ph. sergenti* | *Ph. sergenti* | *Ph. sergenti* |  |
| **185** | Pser23 | *Ph. sergenti* | Male | *Ph. sergenti* | *Ph. sergenti* | *Ph. sergenti* |  |
| **186** | Pser24 | *Ph. sergenti* | Male | *Ph. sergenti* | *Ph. sergenti* | *Ph. sergenti* | PQ868588 |
| **187** | Pser25 | *Ph. sergenti* | Male | *Ph. alexandri* | *Ph. sergenti* | *Ph. sergenti* |  |
| **188** | Pser26 | *Ph. sergenti* | Male | *Ph. jacusieli* | *Ph. sergenti* | *Ph. sergenti* | PQ868589 |
| **189** | Pser27 | *Ph. sergenti* | Male | *Ph. sergenti* | *Ph. sergenti* | *Ph. sergenti* |  |
| **190** | Pser28 | *Ph. sergenti* | Male | *Ph. sergenti* | *Ph. sergenti* | *Ph. sergenti* |  |
| **191** | Psim1 | *Ph. simici* | Female | *Ph. simici* | *Ph. simici* | *Ph. simici* | PQ852124 |
| **192** | Psim2 | *Ph. simici* | Female | *Ph. simici* | *Ph. simici* | *Ph. simici* |  |
| **193** | Psim3 | *Ph. simici* | Female | *Ph. simici* | *Ph. simici* | *Ph. simici* |  |
| **194** | Psim4 | *Ph. simici* | Female | *Ph. syriacus* | *Ph. simici* | *Ph. simici* |  |
| **195** | Psim5 | *Ph. simici* | Female | *Ph. simici* | *Ph. simici* | *Ph. simici* |  |
| **196** | Psim10 | *Ph. simici* | Male | *Ph. syriacus* | *Ph. simici* | *Ph. simici* |  |
| **197** | Psim11 | *Ph. simici* | Male | *Ph. canaaniticus* | *Ph. simici* | *Ph. simici* |  |
| **198** | Psim12 | *Ph. simici* | Male | *Ph. simici* | *Ph. simici* | *Ph. simici* |  |
| **199** | Psim13 | *Ph. simici* | Male | *Ph. simici* | *Ph. simici* | *Ph. simici* |  |
| **200** | Psim14 | *Ph. simici* | Male | *Ph. simici* | *Ph. simici* | *Ph. simici* |  |
| **201** | Psim15 | *Ph. simici* | Male | *Ph. simici* | *Ph. simici* | *Ph. simici* |  |
| **202** | Psim16 | *Ph. simici* | Male | *Ph. simici* | *Ph. simici* | *Ph. simici* |  |
| **203** | Psim17 | *Ph. simici* | Male | *Ph. simici* | *Ph. simici* | *Ph. simici* |  |
| **204** | Psim18 | *Ph. simici* | Male | *Ph. simici* | *Ph. simici* | *Ph. simici* |  |
| **205** | Psim19 | *Ph. simici* | Male | *Ph. simici* | *Ph. simici* | *Ph. simici* |  |
| **206** | Psim20 | *Ph. simici* | Male | *Ph. simici* | *Ph. simici* | *Ph. simici* |  |
| **207** | Psyr1 | *Ph. syriacus* | Female | *Ph. syriacus* | *Ph. syriacus* | *Ph. syriacus* | PQ852123 |
| **208** | Psyr2 | *Ph. syriacus* | Female | *Ph. syriacus* | *Ph. syriacus* | *Ph. syriacus* |  |
| **209** | Psyr3 | *Ph. syriacus* | Female | *Ph. syriacus* | *Ph. syriacus* | *Ph. syriacus* |  |
| **210** | Psyr4 | *Ph. syriacus* | Female | *Ph. syriacus* | *Ph. syriacus* | *Ph. syriacus* |  |
| **211** | Psyr5 | *Ph. syriacus* | Female | *Ph. syriacus* | *Ph. syriacus* | *Ph. syriacus* |  |
| **212** | Psyr6 | *Ph. syriacus* | Female | *Ph. syriacus* | *Ph. syriacus* | *Ph. syriacus* |  |
| **213** | Psyr7 | *Ph. syriacus* | Female | *Ph. syriacus* | *Ph. syriacus* | *Ph. syriacus* |  |
| **214** | Psyr8 | *Ph. syriacus* | Female | *Ph. syriacus* | *Ph. syriacus* | *Ph. syriacus* |  |
| **215** | Psyr9 | *Ph. syriacus* | Female | *Ph. syriacus* | *Ph. syriacus* | *Ph. syriacus* |  |
| **216** | Psyr10 | *Ph. syriacus* | Female | *Ph. syriacus* | *Ph. syriacus* | *Ph. syriacus* |  |
| **217** | Psyr11 | *Ph. syriacus* | Female | *Ph. syriacus* | *Ph. syriacus* | *Ph. syriacus* |  |
| **218** | Psyr12 | *Ph. syriacus* | Female | *Ph. syriacus* | *Ph. syriacus* | *Ph. syriacus* |  |
| **219** | Psyr13 | *Ph. syriacus* | Female | *Ph. syriacus* | *Ph. syriacus* | *Ph. syriacus* |  |
| **220** | Psyr14 | *Ph. syriacus* | Female | *Ph. syriacus* | *Ph. syriacus* | *Ph. syriacus* |  |
| **221** | Psyr15 | *Ph. syriacus* | Female | *Ph. syriacus* | *Ph. syriacus* | *Ph. syriacus* |  |
| **222** | Psyr16 | *Ph. syriacus* | Male | *Ph. syriacus* | *Ph. syriacus* | *Ph. syriacus* |  |
| **223** | Psyr18 | *Ph. syriacus* | Male | *Ph. syriacus* | *Ph. syriacus* | *Ph. syriacus* |  |
| **224** | Psyr19 | *Ph. syriacus* | Male | *Ph. syriacus* | *Ph. syriacus* | *Ph. syriacus* |  |
| **225** | Psyr20 | *Ph. syriacus* | Male | *Ph. syriacus* | *Ph. syriacus* | *Ph. syriacus* |  |
| **226** | Psyr21 | *Ph. syriacus* | Male | *Ph. syriacus* | *Ph. syriacus* | *Ph. syriacus* |  |
| **227** | Psyr24 | *Ph. syriacus* | Male | *Ph. syriacus* | *Ph. syriacus* | *Ph. syriacus* |  |
| **228** | Psyr25 | *Ph. syriacus* | Male | *Ph. syriacus* | *Ph. syriacus* | *Ph. syriacus* |  |
| **229** | Psyr26 | *Ph. syriacus* | Male | *Ph. syriacus* | *Ph. syriacus* | *Ph. syriacus* |  |
| **230** | Psyr27 | *Ph. syriacus* | Male | *Ph. syriacus* | *Ph. syriacus* | *Ph. syriacus* |  |
| **231** | Psyr28 | *Ph. syriacus* | Male | *Ph. syriacus* | *Ph. syriacus* | *Ph. syriacus* |  |
| **232** | Psyr29 | *Ph. syriacus* | Male | *Ph. syriacus* | *Ph. syriacus* | *Ph. syriacus* |  |
| **233** | Ptob2 | *Ph. tobbi* | Female | *Ph. tobbi* | *Ph. tobbi* | *Ph. tobbi* | PQ852125 |
| **234** | Ptob3 | *Ph. tobbi* | Female | *Ph. tobbi* | *Ph. tobbi* | *Ph. tobbi* |  |
| **235** | Ptob5 | *Ph. tobbi* | Female | *Ph. tobbi* | *Ph. tobbi* | *Ph. tobbi* |  |
| **236** | Ptob6 | *Ph. tobbi* | Female | *Ph. tobbi* | *Ph. tobbi* | *Ph. tobbi* |  |
| **237** | Ptob7 | *Ph. tobbi* | Female | *Ph. tobbi* | *Ph. tobbi* | *Ph. tobbi* |  |
| **238** | Ptob8 | *Ph. tobbi* | Female | *Ph. tobbi* | *Ph. tobbi* | *Ph. tobbi* |  |
| **239** | Ptob9 | *Ph. tobbi* | Female | *Ph. tobbi* | *Ph. tobbi* | *Ph. tobbi* |  |
| **240** | Ptob10 | *Ph. tobbi* | Female | *Ph. tobbi* | *Ph. tobbi* | *Ph. tobbi* |  |
| **241** | Ptob11 | *Ph. tobbi* | Female | *Ph. tobbi* | *Ph. tobbi* | *Ph. tobbi* |  |
| **242** | Ptob12 | *Ph. tobbi* | Female | *Ph. tobbi* | *Ph. tobbi* | *Ph. tobbi* |  |
| **243** | Ptob13 | *Ph. tobbi* | Female | *Ph. tobbi* | *Ph. tobbi* | *Ph. tobbi* |  |
| **244** | Ptob14 | *Ph. tobbi* | Female | *Ph. tobbi* | *Ph. tobbi* | *Ph. tobbi* |  |
| **245** | Ptob15 | *Ph. tobbi* | Female | *Ph. tobbi* | *Ph. tobbi* | *Ph. tobbi* |  |
| **246** | Ptob17 | *Ph. tobbi* | Male | *Ph. perfiliewi galilaeus* | *Ph. tobbi* | *Ph. tobbi* |  |
| **247** | Ptob19 | *Ph. tobbi* | Male | *Ph. tobbi* | *Ph. tobbi* | *Ph. tobbi* |  |
| **248** | Ptob20 | *Ph. tobbi* | Male | *Ph. tobbi* | *Ph. tobbi* | *Ph. tobbi* |  |
| **249** | Ptob22 | *Ph. tobbi* | Male | *Ph. tobbi* | *Ph. tobbi* | *Ph. tobbi* |  |
| **250** | Ptob23 | *Ph. tobbi* | Male | *Ph. tobbi* | *Ph. tobbi* | *Ph. tobbi* |  |
| **251** | Ptob24 | *Ph. tobbi* | Male | *Ph. tobbi* | *Ph. tobbi* | *Ph. tobbi* |  |
| **252** | Ptob25 | *Ph. tobbi* | Male | *Ph. tobbi* | *Ph. tobbi* | *Ph. tobbi* |  |
| **253** | Ptob26 | *Ph. tobbi* | Male | *Ph. tobbi* | *Ph. tobbi* | *Ph. tobbi* |  |
| **254** | Ptob27 | *Ph. tobbi* | Male | *Ph. tobbi* | *Ph. tobbi* | *Ph. tobbi* |  |
