# Supplementary material for: A multifaceted molecular approach to surveillance of leishmaniasis: Identification of sand fly species, Leishmania parasites, and blood meal sources using high-resolution melting analysis
Source: PLoS Negl Trop Dis. 2025 Sep 24;19(9):e0013412. doi: 10.1371/journal.pntd.0013412 (PMC12503242; doi:10.1371/journal.pntd.0013412)
Supplement: S2 Table — (DOCX) [file pntd.0013412.s004.docx]

| **Sand fly species (number)** | **Blood meal source** | **Number and percentage of sand fly species which fed on each host** | ***Leishmania* sp. identification in blood meal** |
| --- | --- | --- | --- |
| ***Ph. alexandri***  (n=96) | *Canis lupus familiaris* | 2/96 (2%) | - |
|  | *Equus hemionus* | 27/96 (28%) | - |
|  | *Gazella dorcas* | 10/96 (10%) | - |
|  | *Homo sapiens* | 2/96 (2%) | - |
|  | *Lepus europaeus* | 53/96 (55%) | *L. donovani* (n=3) |
|  | *Vulpes vulpes* | 2/96 (2%) | - |
| ***Ph. arabicus*** (n=2) | *Procavia capensis* | 2/2 (100%) | - |
| ***Ph. halepensis*** (n=3) | *Canis lupus familiaris* | 2/3 (67%) | - |
|  | *Ovis aries* | 1/3 (33%) | - |
| ***Ph. kazeruni*** (n=7) | *Equus asinus* | 4/7 (57%) | - |
|  | *Equus hemionus* | 2/7 (29%) | - |
|  | *Lepus europaeus* | 1/7 (14%) | - |
| ***Ph. papatasi***  (n=110) | *Bos taurus* | 14/110 (13%) | - |
|  | *Canis aureus* | 2/110 (2%) | - |
|  | *Canis lupus familiaris* | 4/110 (3%) | - |
|  | *Capra hircus* | 1/110 (1%) | - |
|  | *Equus caballus* | 5/110 (4.5%) | - |
|  | *Erinaceus concolor* | 10/110 (9%) | - |
|  | *Felis catus* | 4/110 (3%) | - |
|  | *Hemiechinus auritus* | 4/110 (3%) | - |
|  | *Homo sapiens* | 2/110 (2%) | - |
|  | *Lepus europaeus* | 5/110 (4.5%) | - |
|  | *Meles meles* | 1/110 (1%) | - |
|  | *Meriones tristrami* | 25/110 (23%) | *L. major* (n=2) |
|  | *Mus musculus* | 3/110 (3%) | - |
|  | *Ovis aries* | 9/110 (8%) | - |
|  | *Procavia capensis* | 7/110 (6%) | - |
|  | *Psammomys obesus* | 6/110 (5.5%) | - |
|  | *Sus scrofa* | 4/110 (3%) | - |
|  | *Vulpes vulpes* | 4/110 (3%) | - |
| ***Ph. perfiliewi galilaeus***  (n=40) | *Bos taurus* | 22/40 (55%) | - |
|  | *Canis aureus* | 4/40 (10%) | - |
|  | *Canis lupus familiaris* | 2/40 (5%) | - |
|  | *Capra hircus* | 9/40 (22.5%) | - |
|  | *Ovis aries* | 3/40 (7.5%) | - |
| ***Ph. sergenti***  (n=198) | *Alectoris chukar* | 3/198 (1.5%) | - |
|  | *Bos taurus* | 1/198 (0.5%) | - |
|  | *Canis lupus familiaris* | 12/198 (6%) | - |
|  | *Capra hircus* | 1/198 (0.5%) | - |
|  | *Columbia livia* | 1/198 (0.5%) | - |
|  | *Equus asinus* | 1/198 (0.5%) | - |
|  | *Equus hemionus* | 1/198 (0.5%) | - |
|  | *Felis catus* | 76/198 (38%) | - |
|  | *Gazella gazella* | 6/198 (3%) | - |
|  | *Hystrix indica* | 4/198 (2%) | - |
|  | *Lepus europaeus* | 3/198 (1.5%) | - |
|  | *Ovis aries* | 7/198 (3.5%) | - |
|  | *Procavia capensis* | 66/198 (33%) | *L. tropica* (n=1) |
|  | *Sus scrofa* | 2/198 (1%) | - |
|  | *Vulpes vulpes* | 14/198 (7%) | - |
| ***Ph. syriacus*** (n=20) | *Bos taurus* | 12/20 (60%) | - |
|  | *Canis lupus familiaris* | 3/20 (15%) | - |
|  | *Felis catus* | 2/20 (10%) | - |
|  | *Gazella gazella* | 1/20 (5%) | - |
|  | *Sus scrofa* | 2/20 (10%) | - |
| ***Ph. tobbi***  (n=44) | *Bos taurus* | 8/44 (18%) | - |
|  | *Canis aureus* | 3/44 (7%) | - |
|  | *Canis lupus familiaris* | 7/44 (16%) | - |
|  | *Capra hircus* | 6/44 (14%) | - |
|  | *Equus caballus* | 2/44 (4.5%) | - |
|  | *Felis catus* | 7/44 (16%) | - |
|  | *Gazella gazella* | 1/44 (2%) | - |
|  | *Lepus europaeus* | 1/44 (2%) | - |
|  | *Ovis aries* | 2/44 (4.5%) | - |
|  | *Sus scrofa* | 7/44 (16%) | - |
