# Supplementary material for: A multifaceted molecular approach to surveillance of leishmaniasis: Identification of sand fly species, Leishmania parasites, and blood meal sources using high-resolution melting analysis
Source: PLoS Negl Trop Dis. 2025 Sep 24;19(9):e0013412. doi: 10.1371/journal.pntd.0013412 (PMC12503242; doi:10.1371/journal.pntd.0013412)
Supplement: S3 Table — (DOCX) [file pntd.0013412.s005.docx]

**S3 Table.** **Reference amplicon melting temperatures (Tm) obtained by high-resolution melting (HRM) analysis for the identification of Leishmania species, sand fly vectors, and vertebrate blood meal sources.**

Tm values were determined using species-specific HRM-PCR assays targeting the ITS1 region for *Leishmania*, the mitochondrial cytochrome b (*cytb*) gene for sand flies, and *12S* and *16S* for blood meal source identification. These values serve as diagnostic reference profiles for species differentiation in HRM-based molecular analyses.

| **Species** | **Tm** |
| --- | --- |
| ***Leishmania* species** | |
| ***L. major*** | 78.52 |
| ***L. tropica*** | 77.98 |
| ***L. infantum*** | 80.42 |
| ***L. donovani*** | 80.60 |
| **Sand fly species** | |
| ***Ph. alexandri*** | 72.15 |
| ***Ph. arabicus*** | 72.96 |
| ***Ph. canaaniticus*** | 71.95 |
| ***Ph. halepensis*** | 73.65 |
| ***Ph. jacusieli*** | 71.99 |
| ***Ph. kazeruni*** | 73.92 |
| ***Ph. papatasi*** | 75.04 |
| ***Ph. perfiliewi galilaeus*** | 73.23 |
| ***Ph. sergenti*** | 76.42 |
| ***Ph. simici*** | 72.05 |
| ***Ph. syriacus*** | 70.91 |
| ***Ph. tobbi*** | 72.47 |
| **Blood meal source** | |
| **Chukar partridge** (*Alectoris chukar*) | 82.90 |
| **Cow** (*Bos taurus*) | 81.32 |
| **Golden jackal** (*Canis aureus*) | 81.18 |
| **Domestic dog** (*Canis lupus familiaris*) | 81.19 |
| **Goat** (*Capra hircus*) | 80.85 |
| **Rock dove** (*Columba livia*) | 82.73 |
| **Donkey** (*Equus asinus*) | 81.18 |
| **Horse** (*Equus caballus*) | 80.59 |
| **Onager** (*Equus hemionus*) | 81.26 |
| **Hedgehog** (*Erinaceus concolor*) | 77.98 |
| **Domestic cat** (*Felis catus*) | 81.40 |
| **Dorcas gazelle** (*Gazella dorcas*) | 79.80 |
| **Mountain gazelle** (*Gazella gazella*) | 80.50 |
| **Long-eared hedgehog** (*Hemiechinus auritus*) | 79.88 |
| **Human** (*Homo sapiens*) | 82.74 |
| **Indian crested porcupine** (*Hystrix indica*) | 81.62 |
| **European hare** (*Lepus europaeus*) | 78.91 |
| **European badger** (*Meles meles*) | 80.67 |
| **Tristram’s jird** (*Meriones tristrami*) | 82.23 |
| **House mouse** (*Mus musculus*) | 79.92 |
| **Domestic sheep** (*Ovis aries*) | 79.50 |
| **Rock hyrax** (*Procavia capensis*) | 82.43 |
| **Fat sand rat** (*Psammomys obesus*) | 80.49 |
| **Wild boar** (*Sus scrofa*) | 81.02 |
| **Red fox** (*Vulpes vulpes*) | 80.86 |
