# Supplementary material for: A multifaceted molecular approach to surveillance of leishmaniasis: Identification of sand fly species, Leishmania parasites, and blood meal sources using high-resolution melting analysis
Source: PLoS Negl Trop Dis. 2025 Sep 24;19(9):e0013412. doi: 10.1371/journal.pntd.0013412 (PMC12503242; doi:10.1371/journal.pntd.0013412)
Supplement: S1 File — (DOCX) [file pntd.0013412.s006.docx]

**Supplementary Information S3 – Multiple Sequence Alignments for HRM Targets**

This document provides the multiple sequence alignments used to support the interpretation of High-Resolution Melt (HRM) profiles presented in this study. These alignments demonstrate the genetic variation among target sequences and help explain the HRM peak differences observed for pathogen detection, vector identification, and blood meal analysis.

All alignments were generated using the CLUSTALW online tool (default parameters). Three alignment sets are included:

***Leishmania* ITS1 sequences**
Four reference sequences were selected from species present in Israel: *L. major*, *L. tropica*, *L. infantum*, and *L. donovani*. These were used to assess species-level variation relevant to parasite detection.

**Sand fly *cytb* sequences**
A total of 28 sequences representing 12 sand fly species were aligned to highlight inter-species differences in mitochondrial DNA, which convey vector discrimination via HRM.

**Vertebrate *12S*/*16S* *rRNA* blood meal sequences**
Twenty-five vertebrate sequences were included, corresponding to the potential blood meal sources identified in the study. This alignment supports the resolution of host species via HRM.

These data provide a molecular basis for the HRM profiles reported in the results and are referenced in the response to reviewer comments.

***Leishmania* species multiple sequence alignment**

A total 4 sequences of 4 *Leishmania* species: *L. major* (MZ366759), *L. tropica* (MZ366759), *L. infantum* (MW534746), and *L. donovani* (MW534748).

MW534746.1 TTCCGATGATTACACCCAAAAAACATATACAACTCGGGGAGACCTATGTATATATATGTA

MZ366760.1 --------------CCTATATATTATACATTATATAGG----------------------

MW534748.1 -----------------ATGTATATATATATATGTAGG----------------------

MZ366759.1 -------------TTATTCTATATATATATAGTATAGG----------------------

: ::: ::: : :. .**

MW534746.1 GGCCTTTCCCACATACACAGCAAAGTTTTGTACTCAAAATTTGCAGTAAAAAAAAGGCCG

MZ366760.1 CCTTTCCCACACATACACAGCAAACTTTTATACTCGAAGTTTGCAGTAAACAAAAGGCCG

MW534748.1 --CCTTTCCCACATACACAGCAAAGTTTTGTACTCAAAATTTGCAGTAAAAAAAAGGCCG

MZ366759.1 --CTTTTCCCACATACACAGCAAACTTTTATACTCGAAATTTGCAG--TAAAAAAGGCCG

* *.*************** ****.*****.**.******* :*.*********

MW534746.1 ATCGACGTT--ATAACGCACCGCCTAT--ACAAAAGCAAAAATGTCCGTTTATACAAAAA

MZ366760.1 ATCGACCTT--ATAACGCACCGCCTATACACAAAAGCAAAAATGTCCGTTTATACAAATA

MW534748.1 ATCGACGTT--ATAACGCACCGCCTAT--ACAAAAGCAAAAATGTCCGTTTATACAAAAA

MZ366759.1 ATCGACGTTGTAGAACGCACCGCCTATACACAAAAGCAAAAATGTCCGTTTATACAAAAA

****** ** * ************** *****************************:*

MW534746.1 ATAT-ACGGCGTTTCGGTTTT--------------TGGCGGGGTGGGTGCGTGTGTGGAT

MZ366760.1 T----ACGGCGTTTCGGTTTTG------------TTGGCGGGGGGTGCGTGTGTGTGGAT

MW534748.1 ATAT-ACGGCGTTTCGGTTTT--------------TGGCGGGGTGGGTGCGTGTGTGGAT

MZ366759.1 AATAGACGGCGTTTCGGTTTTTGGCGGGAGGGAGAGAGAGGGGGGTGCGTGCGCGTGGAT

: **************** .*.**** * * * * * ******

MW534746.1 AACGGCTCACATAACGTGTCGCGATGGATGACTTGGCTTCCTATTTCGTTGAAGAACGCA

MZ366760.1 AACGGCTCACATAACGTGTCGCGATGGATGACTTGGCTTCCTATTTCGTTGAAGAACGCA

MW534748.1 AACGGCTCACATAACGTGTCGCGATGGATGACTTGGCTTCCTATTTCGTTGAAGAACGCA

MZ366759.1 AACGGCTCACATAACGTGTCGCGATGGATGACTTGGCTTCCTATTTCGTTGAAGAACGCA

************************************************************

MW534746.1 GTAAAGTGCGATAAGTGGTATCAATTGCAGAATCATTCAATTACCGAATCTTTGAACGCA

MZ366760.1 GTAAAGTGCGATAAGTGGTATCAATTGCAGAATCATTCAATTACCGAATCTTTGAACGCA

MW534748.1 GTAAAGTGCGATAAGTGGTATCAATTGCAGAATCATTCAATTACCGAATCTTTGAACGCA

MZ366759.1 GTAAAGTGCGATAAGTGGTATCAATTGCAGAATCATTCAATTACCGAATCTTTGAACGCA

************************************************************

MW534746.1 AACGGCGCATGGGAGAAGCTCTATTGTGTCATCCCCGTGCATGCCATATTCTCAGTGTCG

MZ366760.1 AACGGCGCATGGGAGAAGCTCTATTGTGTCATCCCCGTGCATGCCATATTCTCAGTGTCG

MW534748.1 AACGGCGCATGGGAGAAGCTCTATTGTGTCATCCCCGTGCATGCCATATTCTCAGTGTCG

MZ366759.1 AACGGCGCATGGGAGAAGCTCTATTGTGTCATCCCCGTGCATGCCATATTCTCAGTGTCG

************************************************************

MW534746.1 AACAAAAAACAACACGCCGCCTCCTCTCTTCTGCACATATATATATTATACCATACACAG

MZ366760.1 AACAAAAAACAACACGCCGCCTCCTCTCTTCTGCATATATGTATAT------ATATACAT

MW534748.1 AACAAAAAACAACACGCCGCCTCCTCTCTTCTGCACATATATATATTATACCATACACAG

MZ366759.1 AACAAAAAACAACACGCCGCCTCCTCTCTTCTGCATATATTTATAT------ATATACAA

*********************************** **** ***** *** ***

MW534746.1 TATATATATAATTATGTGTTGGAAGCCAAGAGGAGGCGTGTGTTTGTGTTGTGCGCATAT

MZ366760.1 TATATATAATATGTG----TGGAAGCCAAGAGGAGGCGTGTGTTTGTGTTGGGCGCATAT

MW534748.1 TATATATATAATTATGTGTTGGAAGCCAAGAGGAGGCGTGTGTTTGTGTTGTGCGCATAT

MZ366759.1 TATATAATATATGTG----TGGAAGCCAAGAGGAGGCGTGTGTTTGTGTTGTGCCCATAT

******::::** : ******************************** ** *****

MW534746.1 TATATGTATATATG---CTGTGTGCACACGTAGACAAGTTAGAGTTGGACAAATACACAC

MZ366760.1 ATATATATATTATATGTGTGTGTGCACACGTAGACAAGTTAGAGTTGGACAAATACACGC

MW534748.1 TATATGTATATATG---CTGTGTGCACACGTAGACAAGTTAGAGTTGGACAAATACACAC

MZ366759.1 ATATATATAT-------GTGTGTGCACACGTAGACAAGTCAGAGTTGGACAAATACACAC

::::: :::: ********************* ******************.*

MW534746.1 ATG----CACTCTCTTTTGTGTGGGTGCGCGCGTGGAAACTCCTCTCTGGTGCTTGCAAA

MZ366760.1 ATG----CACTCTCTTTTGTGTGGGTGCGAGCGTCGAAACTCCTCTCTGGTGCTTGCGCA

MW534748.1 ATG----CACTCTCTTTTGTGTGGGTGCGCGCGTGGAAACTCCTCTCTGGTGCTTGCAAA

MZ366759.1 GCGTGTGCACTCTCTTTTGTGTGCGTGCGCGCGTGGAAACTCCTCTCTGGTGCTTGCAAA

. * **************** *****.**** **********************..*

MW534746.1 GCAGTCTTTTTCTCTTTCTCT-----------TTTTCTCTCTCCATTCTCTCCTCTCTTT

MZ366760.1 AAGCAGTCTTTCCCTTTCTCT-----------CTTTCTCTCTCCATTTTCTCTCCTCTTT

MW534748.1 GCAGTCTTTTTCTCTTTCTCT-----------TTTTCTCTCTCCATTCTCTCCTCTCTTT

MZ366759.1 AAGCAGTCTTTCCCTTTCTCCTTCTCCTTTCTCTTTCTCTCTCCATTCTCTCCTCTCTTT

... : * **** ******* ************** **** ******

MW534746.1 TTT-CATCAAAAAGGGGGGAGAGAAAAAGAGAG----------AGGAGGGGGGGTCGAGG

MZ366760.1 TTT-CATCAAAAAAGGGGGAGAGAAAAAAGAGGGAGA------AAAAGGGGGTCGAGAGG

MW534748.1 TTT-CATCAAAAAGGGGGGAGAGAAAAAGAGAG------------AGGAGGGGGTCGAGG

MZ366759.1 TTTTCATCAAAAAGGGGGGAGAGAAAAAGAGGGAGAAGAAGACACGCGGGAGTCGCGAGG

*** *********.**************....* . *.*.* .****

MW534746.1 --GAGAGAGGCTGTGACCAGGATTATTAAACAAAAAACCAAAACGAGAATTCAACTTCGC

MZ366760.1 --GAGAGAGGCTGTGACCAGGATTATTAAACAAAAAACCAAAACGAGAATTCAACTTCGC

MW534748.1 --GAGAGAGGCTGTGACCAGGATTATTAAACAAAAAACCAAAACGAGAATTCAACTTCGC

MZ366759.1 GAGAGAGAGGCTGTGATTAGGATTATTAAACAAAAAACCAAAACGAGAATTCAACTTCGC

************** ******************************************

MW534746.1 GTTGGCCATTTTTTGCTTAATGGGGGG-AGGTGGGTGTGGGTGGTGTGTGGCTCTCTCTC

MZ366760.1 GTTGGCCATTTTTTGCTTAATGGGGGG-TGGG----------------------------

MW534748.1 GTTGGCCATTTTTTGCTTAATGGGGGGGAGGTGGGTGTGGGTGGTGTGTGGCTCTCTCTC

MZ366759.1 GTTGGCCATTTTTTGCTTAATGGGGGGTGGG-----------------------------

*************************** **

MW534746.1 TGTGTGGTATATATATATGTATATTAGAAGTAGGTTGTGTGTGTGTGTATGTGTTTTACA

MZ366760.1 ------------------------------------------------------------

MW534748.1 TGTGTGGTATATATATATGTATATTAGAAGTAGGTTGTGTGTGTGTGTATGTGTTTTACA

MZ366759.1 ------------------------------------------------------------

MW534746.1 CATATATATATCCGCGCCCTCACTCTCTCATATATAATTTATATGTACGCACAGAGAAAA

MZ366760.1 ------------------------------------------------------------

MW534748.1 CATATATATATCCGCGCCCTCACTCTCTCATATATAATTTATATGTACGCACAGAGAAAA

MZ366759.1 ------------------------------------------------------------

MW534746.1 AAGAGAGGCGCTCTCTTTTCCCCCCACCCCCGACAACCTTTGTTTACAGACCTGA

MZ366760.1 -------------------------------------------------------

MW534748.1 AAGAGAGGCGCTCTCTTTTCCCCCCACCCCCGACAACCTTTGTTTACAGACCT--

MZ366759.1 -------------------------------------------------------

**Sand fly species multiple sequence alignment** (according to GenBank accession numbers in the SI_S1 Table)

A total 28 sequences of 12 sand fly species: *Ph. alexandri* (PQ868583, PQ868584), *Ph. arabicus* (PQ852116), *Ph. canaaniticus* (PQ815623), *Ph. halepensis* (PQ852115), *Ph. jacusieli* (PQ868585), *Ph. kazeruni* (PQ852118, PQ868586, PQ852119), *Ph. papatasi* (PQ868587, PQ852120, PQ852121), *Ph. perfiliewi galilaeus* (PQ852131, PQ852128, PQ852132, PQ852135, PQ852126, PQ852134, PQ852130, PQ852133, PQ852127), *Ph. sergenti* (PQ868589, PQ852122, PQ868588), *Ph. simici* (PQ852124), *Ph. syriacus* (PQ852123), *Ph. tobbi* (PQ852125).

PQ868583.1 ------------------ATATCAATTGCTATCTTATTTTTAATACCTATTCTTCATGTT

PQ868584.1 ------------------ATATCAATTGCTATCTTATTTTTAATACCTATTCTTCATGTT

PQ852128.1 GGAGTAATCGCCCTTGTTATATCAATTGCTATTTTATTTATTCTACCAATTTTACATGTT

PQ852132.1 ------------------ATATCAATTGCTATTTTATTTATTCTACCAATTTTACATGTT

PQ852125.1 ----------------TTATATCAATTGCTATTTTATTTATTTTACCAATTTTACATACT

PQ852131.1 ---------------GTTATATCAATTGCTATTTTATTTATTCTACCAATTTTACATGTT

PQ852135.1 ---------------GTTATATCAATTGCTATTTTATTTATTCTACCAATTTTACATGTT

PQ852133.1 ------------------ATATCAATTGCTATTTTATTTATTCTACCAATTTTACATGTT

PQ852130.1 ------------------ATATCAATTGCTATTTTATTTATTCTACCAATTTTACATGTT

PQ852122.1 --------------------ATCTATTGCAATTCTATTTTTAATACCCCTTTTACACACT

PQ868588.1 ------------------------------------TTTTTAATACCCCTTTTACACACT

PQ868589.1 -------------------TATCTATTGCAATTCTATTTTTAATACCCCTTTTACACACT

PQ852127.1 ------------------ATATCAATTGCTATTTTATTTATTTTACCAATTTTACATGTT

PQ852134.1 ------------------ATATCAATTGCTATTTTATTTATTCTACCAATTTTACATGTT

PQ852126.1 ---------------GTTATATCAATTGCTATTTTATTTATTTTACCAATTTTACATGTT

PQ815623.1 ---------------------TCTATTGCTATTTTATTTATTTTACCTATTTTACACATA

PQ868585.1 ------------------ATATCTATTGCAATTCTATTTCTAATACCTTTTTTACATACT

PQ852118.1 ---------------GTTATATCTATTGCAATTTTATTTTTAATACCTTTTCTACATACA

PQ868586.1 ---------------------TCTATTGCAATTTTATTTTTAATACCTTTTCTACATACA

PQ852119.1 ---------------------TCTATTGCAATTTTATTTTTAATACCTTTTCTACATACA

PQ868587.1 ---------------GTTATATCAATTGCTATCCTTTTCCTTATACCTTTACTCCATACA

PQ852120.1 ------------------ATATCAATTGCTATCCTTTTCCTTATACCTTTACTCCATACA

PQ852121.1 ------------------ATATCAATTGCTATCCTTTTCCTTATACCTTTACTCCATACA

PQ852123.1 ---------------GTTATATCAATTGCAATTTTATTTATTCTTCCTTTATTACATATA

PQ852116.1 ---------------------TCTATTGCAATCCTGTTTATTCTACCTATTCTACATATA

PQ852117.1 ---------------------TCTATTGCAATCCTATTTATTCTACCTATTCTACATATA

PQ852115.1 ------------------ATATCTATTGCAATCCTATTTATTCTACCTATTCTACACATA

PQ852124.1 ----------------TTATATCTATTGCTATTTTATTTATTTTACCTATTTTACATGTA

** *: *:** *: * ** . :

PQ868583.1 AATCAATCTCAAGGATTACAATTTTATCCATTAAATCAAATTTTATTTTGATATATAGTA

PQ868584.1 AATCAATCTCAAGGATTACAATTTTATCCATTAAATCAAATTTTATTTTGATATATAGTA

PQ852128.1 AGCAAAACACAAGGGTTACAATTTTATCCTATTAATCAAATTTTATTTTGATATATAGTT

PQ852132.1 AGCAAAACACAAGGATTACAATTTTATCCTATTAATCAAATTTTATTTTGATATATAGTT

PQ852125.1 AATAAATCACAAGGATTACAATTTTATCCTATCAATCAAATTTTATTTTGATATATAGTT

PQ852131.1 AGCAAAACACAAGGGTTACAATTTTATCCTATTAATCAAATTTTATTTTGATATATAGTT

PQ852135.1 AGCAAAACACAAGGGTTACAATTTTATCCTATTAATCAAATTTTATTTTGATATATAGTT

PQ852133.1 AGCAAAACACAAGGATTACAATTTTATCCTATTAATCAAATTTTATTTTGATATATAGTT

PQ852130.1 AGCAAAACACAAGGGTTACAATTTTATCCTATTAATCAAATTTTATTTTGATATATAGTT

PQ852122.1 AACGAAACCCGGGGTCTTCAATTTTACCCACTAAATCAAATTTTATTCTGATATATAGTA

PQ868588.1 AACGAAACCCGAGGTCTTCAATTTTACCCACTAAATCAAATTTTATTCTGATATATAGTA

PQ868589.1 AACGAAACCCGGGGTCTTCAATTTTACCCACTAAATCAAATTTTATTCTGATATATAGTA

PQ852127.1 AGCAAAACACAAGGGTTACAATTTTATCCTATTAATCAAATTTTATTTTGATATATAGTT

PQ852134.1 AGCAAAACACAAGGATTACAATTTTATCCTATTAATCAAATTTTATTTTGATATATAGTT

PQ852126.1 AGCAAAACACAAGGGTTACAATTTTATCCTATTAATCAAATTTTATTTTGATATATAGTT

PQ815623.1 AGTAAATTTCAAGGATTACAATTCTACCCTTTAAATCAAATTTTATTTTGATATATAGTT

PQ868585.1 AACCAAACTCAAGGCCTCCAATTTTATCCATTAAATCAAATTTTATTTTGATATATAGTT

PQ852118.1 AATGAAACACAAGGGCTTCAATTTTATCCTTTAAATCAAATTTTATTTTGATATATAGTT

PQ868586.1 AATGAAACACAAGGACTTCAATTTTATCCTTTAAATCAAATTTTATTTTGATATATAGTT

PQ852119.1 AATGAAACACAAGGGCTTCAATTTTATCCTTTAAATCAAATTTTATTTTGATATATAGTT

PQ868587.1 AATCAATCACAAGGACTTCAATTTTACCCGTTAAATCAAATCCTATTCTGATATATAGTA

PQ852120.1 AATCAATCACAAGGACTTCAATTTTACCCATTAAATCAAATCCTATTCTGATATATAGTA

PQ852121.1 AATCAATCACAAGGACTTCAATTTTACCCATTAAATCAAATCCTATTCTGATATATAGTA

PQ852123.1 AATAAATCACAAGGATTACAATTTTACCCAGTTAATCAAGTATTATTTTGATATATAGTT

PQ852116.1 AATAAATTTCAAGGACTCCAATTTTATCCTCTAAATCAAATTTTATTTTGATATATAGTA

PQ852117.1 AATAAATTTCAAGGACTCCAATTTTATCCTCTAAATCAAATTTTATTTTGATATATAGTA

PQ852115.1 AATAAATTTCAAGGACTTCAATTTTATCCTCTAAATCAAATTTTATTTTGATATATAGTT

PQ852124.1 AGTAAATTTCAAGGACTCCAATTCTATCCAATTAATCAAGTATTATTTTGATATATAGTT

*. **: *..** * ***** ** ** * ******.* **** ***********:

PQ868583.1 ATTACAATTATTCTTTTAACATGAATTGGTGCTCGACCTGTTGAAACTCCTTATATTTTA

PQ868584.1 ATTACAATTATTCTTTTAACATGAATTGGTGCTCGACCTGTTGAAACTCCTTATATTTTA

PQ852128.1 ATTATTATTGTTTTATTAACATGAATTGGAGCCCGTCCCGTCGAAGCCCCATTTATTTTA

PQ852132.1 ATTATTATTGTTTTATTAACATGAATTGGAGCCCGTCCCGTCGAAGCCCCATTTATTTTA

PQ852125.1 ATTATTATTGTTTTATTAACATGAATTGGAGCCCGCCCTGTTGAAGCTCCTTTTATTTTA

PQ852131.1 ATTATTATTATTTTATTAACATGAATTGGAGCCCGTCCCGTCGAAGCCCCATTTATTTTA

PQ852135.1 ATTATTATTATTTTATTAACATGAATTGGAGCCCGTCCCGTCGAAGCCCCATTTATTTTA

PQ852133.1 ATTATTATTATTTTATTAACATGAATTGGAGCCCGTCCCGTCGAAGCCCCATTTATTTTA

PQ852130.1 ATTATTATTGTTTTATTAACATGAATTGGAGCCCGTCCCGTCGAAGCCCCATTTATTTTA

PQ852122.1 ATTATAATTCTTCTATTGACTTGAATTGGAGCCCGTCCAGTAGAAGCTCCCTATATCATT

PQ868588.1 ATTATAATTCTTCTATTGACTTGAATTGGGGCCCGTCCAGTAGAAGCTCCCTATATCATT

PQ868589.1 ATTATAATTCTTCTATTGACTTGAATTGGGGCCCGTCCAGTAGAAGCTCCCTATATCATT

PQ852127.1 ATTATTATTGTTTTATTAACATGAATTGGAGCCCGTCCCGTCGAAGCCCCATTTATTTTA

PQ852134.1 ATTATTATTGTTTTATTAACATGAATTGGAGCCCGTCCCGTCGAAGCCCCATTTATTTTA

PQ852126.1 ATTATTATTGTTTTATTAACATGAATTGGAGCCCGTCCCGTCGAAGCCCCATTTATTTTA

PQ815623.1 ATTATTATTATATTACTTACATGAATTGGGGCTCGCCCTGTTGAAGCACCATATATTTTA

PQ868585.1 ATTATAATTCTCTTATTAACTTGAATTGGAGCCCGCCCCGTAGAAACCCCTTATATTCTT

PQ852118.1 ATTATAATTTTACTTCTAACTTGAATTGGAGCACGCCCAGTTGAGGCCCCTTATATTATT

PQ868586.1 ATTATAATTTTACTTCTAACTTGAATTGGAGCACGCCCAGTTGAGGCCCCTTATATTATT

PQ852119.1 ATTATAATTTTACTTCTAACTTGAATTGGGGCACGCCCAGTTGAGGCCCCTTATATTATT

PQ868587.1 ATTACTATTATTCTATTAACATGAATCGGAGCTCGTCCTGTTGAAACTCCTTATATTTTA

PQ852120.1 ATTACTATTATTCTATTAACATGAATCGGAGCTCGTCCTGTTGAAACTCCTTATATTTTA

PQ852121.1 ATTACTATTATTCTATTAACATGAATCGGAGCTCGTCCTGTTGAAACTCCTTATATTTTA

PQ852123.1 ATTATTATTATTCTATTAACATGAATTGGAGCACGTCCAGTTGAAGCCCCTTTTATTCTC

PQ852116.1 ATTATTATTATATTATTAACATGAATTGGAGCTCGTCCTGTTGAAACACCTTATATTCTT

PQ852117.1 ATTATTATTATATTATTAACATGAATTGGAGCTCGTCCTGTTGAAACACCTTATATTCTT

PQ852115.1 ATTATTATTGTATTATTAACATGAATTGGAGCTCGTCCTGTTGAAACACCTTATATTCTT

PQ852124.1 ATTATTATTATTTTATTAACATGAATTGGAGCTCGTCCTGTTGAAACTCCATATATTTTA

**** :*** * *: * **:***** ** ** ** ** ** **..* ** *:*** *

PQ868583.1 ACTGGCCAAATTTTAACTGTTTTATATTTTTCTTATTATATTTTAAATCCCCTTATCTCT

PQ868584.1 ACTGGCCAAATTTTAACTGTCTTATATTTTTCTTATTATATTTTAAATCCTCTTATCTCT

PQ852128.1 ACTGGTCAAATTCTTACAGTCCTTTATTTCTCTTATTATATTTTAAATCCTATAATTTCA

PQ852132.1 ACTGGTCAAATTCTTACAGTCCTTTATTTCTCTTATTATATTTTAAATCCTATAATTTCA

PQ852125.1 ACAGGACAAATTTTAACTGTTCTTTACTTCTCTTATTATATTCTAAATCCTATAATTTCA

PQ852131.1 ACTGGTCAAATTCTTACAGTCCTTTATTTCTCTTATTATATTTTAAATCCTATAATTTCA

PQ852135.1 ACTGGTCAAATTCTTACAGTCCTTTATTTCTCTTATTATATTTTAAATCCTATAATTTCA

PQ852133.1 ACTGGTCAAATTCTTACAGTCCTTTATTTCTCTTATTATATTTTAAATCCTATAATTTCA

PQ852130.1 ACTGGTCAAATTCTTACAGTCCTTTATTTCTCTTATTATATTTTAAATCCTATAATTTCA

PQ852122.1 ACTGGTCAAATTTTAACTATTCTTTATTTCTCCTACTATATTATTAACCCTTTAATTATC

PQ868588.1 ACTGGTCAAATTTTAACTATTCTTTATTTCTCCTACTATATTATTAACCCTTTAATTATC

PQ868589.1 ACTGGTCAAATTTTAACTATTCTTTATTTCTCCTACTATATTATTAACCCTTTAATTATC

PQ852127.1 ACTGGTCAAATTCTTACAGTTCTTTATTTCTCTTATTATATTTTAAATCCTATAATTTCA

PQ852134.1 ACTGGTCAAATTCTTACAGTCCTTTATTTCTCTTATTATATTTTAAATCCTATAATTTCA

PQ852126.1 ACTGGTCAAATTCTTACAGTCCTTTATTTCTCTTATTATATTTTAAATCCTATAATTTCA

PQ815623.1 ACTGGTCAAATTCTAACAGTTCTTTATTTTTCTTATTATATTTTAAATCCTATAATTTCT

PQ868585.1 ACCGGTCAAATTCTTACTGTCCTTTACTTTTCTTATTATATTATTAATCCTATAATTATC

PQ852118.1 ACAGGACAAATTTTAACTGTCCTTTATTTTTCCTATTACATTCTTAATCCAATAATTACA

PQ868586.1 ACAGGACAAATTTTAACTGTCCTTTATTTTTCCTATTACATTCTTAATCCAATAATTACA

PQ852119.1 ACAGGACAAATTTTAACTGTCCTTTATTTTTCCTATTACATTCTTAATCCAATAATTACA

PQ868587.1 ACAGGACAAATTTTAACTGTTCTTTACTTCTCTTATTATATTTTAAATCCAATTATTTCA

PQ852120.1 ACAGGACAAATTTTAACTGTTCTTTACTTCTCTTATTATATTTTAAATCCAATTATTTCA

PQ852121.1 ACAGGACAAATTTTAACTGTTCTTTACTTCTCTTATTATATTTTAAATCCAATTATTTCA

PQ852123.1 ACAGGTCAAATTTTAACAGTTCTTTATTTTTCTTATTATTTAATAAATCCAATAATCTCA

PQ852116.1 ACAGGTCAAATTTTAACTGTTCTCTACTTTTCTTACTATATTATAAACCCTATAATTTCT

PQ852117.1 ACAGGTCAAATTTTAACTGTTCTCTACTTTTCTTATTATATTATAAACCCTATAATTTCT

PQ852115.1 ACAGGTCAAATTTTAACTGTTCTCTATTTTTCTTACTATCTTATAAACCCTATAATTTCT

PQ852124.1 ACAGGTCAAATCTTAACTGTTCTTTATTTTTTTTATTATATCATAAACCCAATCATTTCT

** ** ***** *:**:.* * ** ** * ** ** * *:** ** * ** :

PQ868583.1 AAACTATGAGATAATTT--------ATTAA----------------ATAATTAGTTAATG

PQ868584.1 AAACTATGAGATAATTT--------ATTAA----------------ATAATTAGTTAATG

PQ852128.1 AAAATTTGAGATAATTTTCTAAAATAACCT----------------CTAACTAGTTAATG

PQ852132.1 AAAATTTGAGATAATTTTCTAAAATAACCT----------------CTAACTAGTTAATG

PQ852125.1 AAAATTTGAGATAATTTTCTAAAATAATCT----------------T-ATTTAGTTAATG

PQ852131.1 AAAATTTGAGATAATTTTCTAAAATAACCT----------------CTAACTAGTTAATG

PQ852135.1 AAAATTTGAGATAATTTTCTAAAATAACCT----------------CTAACTAGTTAATG

PQ852133.1 AAAATTTGAGATAATTTTCTAAAATAACCT----------------CTAACTAGTTAATG

PQ852130.1 AAAATTTGAGATAATTTTCTAAAATAACCT----------------CTAACTAGTTAATG

PQ852122.1 AAATTATGAGACAATAT----ATTAAACTC----------------ATAATAAGTTAATA

PQ868588.1 AAATTATGAGACAATAT----ATTAAACTC----------------ATAATAAGTTAATA

PQ868589.1 AAATTATGAGACAATAT----ATTAAACTC----------------ATAATAAGTTAATA

PQ852127.1 AAAATTTGAGATAATTTTCTAAAATAACCT----------------CTAACTAGTTAATG

PQ852134.1 AAAATTTGAGATAATTTTCTAAAATAACCT----------------CTAACTAGTTAATG

PQ852126.1 AAAATTTGAGATAATTTTCTAAAATAACCT----------------CTAACTAGTTAATG

PQ815623.1 AAAATCTGAGATAAACT--------ATTAA----------------ACTATTAGTTAATA

PQ868585.1 AAATTATGAGACAATCT----ATTAAACTC----------------ATAATAAGTTAATA

PQ852118.1 AAATTATGAGATTATCT----ATTAAACTC----------------ATAAATAGTTAATA

PQ868586.1 AAATTATGAGATTATCT----ATTAAACTC----------------ATAAATAGTTAATA

PQ852119.1 AAATTATGAGATTATCT----ATTAAACTC----------------ATAAATAGTTAATA

PQ868587.1 AAATTCTGAGATAACTT----ATTAAAT--------------------AATTAGTTAATA

PQ852120.1 AAATTCTGAGATAACTT----ATTAAAT--------------------AATTAGTTAATA

PQ852121.1 AAATTCTGAGATAACTT----ATTAAAT--------------------AATTAGTTAATA

PQ852123.1 AAAATTTGAGATAATCTTCTAAAATAATATTAACAATTATATTAATTAAATTAGTTAATG

PQ852116.1 AAAATCTGAGATAAATT----ACTAAACTA----------------CTAG----TTAATA

PQ852117.1 AAAATCTGAGATAAATT----ACTAAACTA----------------CTAG----TTAATA

PQ852115.1 AAAATCTGAGACAAATT----ACTAAACTA----------------TTAG----TTAATA

PQ852124.1 AAAATTTGAGATAAATT----ATTAAATTA----------------TTAATTAGTTAATA

*** * ***** :* * *: : *****.

PQ868583.1 AGCTTGAATAG-CAATTGTTTTGAAAACATTAGATAGAAACTAAAATTTTCTATTAACTT

PQ868584.1 AGCTTGAATAG-CAATTGTTTTGAAAACATTAGATAGAAACTAAAATTTTCTATTAACTT

PQ852128.1 AGCTTGAAAAG-CAATTGTTTTGAAAACATTAGATAGAAACTAAAATTTTCTATTAACTT

PQ852132.1 AGCTTGAAAAG-CAATTGTTTTGAAAACATTAGATAGAAATTAAAATTTTCTATTAACTT

PQ852125.1 AGCTTGAACAG-CAATTGTTTTGAAAACATTAGATAGAAACTAAAATTTTCTATTAACTT

PQ852131.1 AGCTTGAAAAG-CAATTGTTTTGAAAACATTAGATAGAAACTAAAATTTTCTATTAACTT

PQ852135.1 AGCTTGAAAAG-CAATTGTTTTGAAAACATTAGATAGAAATTAAAATTTTCTATTAACTT

PQ852133.1 AGCTTGAAAAG-CAATTGTTTTGAAAACATTAGATAGAAACTAAAATTTTCTATTAACTT

PQ852130.1 AGCTTGAAAAG-CAATTGTTTTGAAAACATTAGATAGAAATTAAAATTTTCTATTAACTT

PQ852122.1 AGCTTAAATAG-CAATTGTTTTGAAAACATTAGATAGAAATTAAAGCTTTCTATTAACTT

PQ868588.1 AGCTTAAATAG-CAATTGTTTTGAAAACATTAGATAGAAATTAAAGCTTTCTATTAACTT

PQ868589.1 AGCTTAAATAG-CAATTGTTTTGAAAACATTAGATAGAAATTAAAGCTTTCTATTAACTT

PQ852127.1 AGCTTGAAAAG-CAATTGTTTTGAAAACATTAGATAGAAACTAAAATTTTCTATTAACTT

PQ852134.1 AGCTTGAAAAG-CAATTGTTTTGAAAACATTAGATAGAAACTAAAATTTTCTATTAACTT

PQ852126.1 AGCTTGAAAAG-CAATTGTTTTGAAAACATTAGATAGAAACTAAAATTTTCTATTAACTT

PQ815623.1 AGCTTAAATAAGCAATTGTTTTGAAAACATTAGATAGAAATTAAAACTTTCTATTAACTT

PQ868585.1 AGCTTAAATAG-CAATTGTTTTGAAAACATTAGATAGAAATTAAAATTTTCTATTAACTT

PQ852118.1 AGCTTTAATAG-CAATTGTTTTGAAAACATTAGATAGAAATTAAAACTTTCTATTAACTT

PQ868586.1 AGCTTTAATAG-CAATTGTTTTGAAAACATTAGATAGAAATTAAAACTTTCTATTAACTT

PQ852119.1 AGCTTTAATAG-CAATTGTTTTGAAAACATTAGATAGAAATTAAAACTTTCTATTAACTT

PQ868587.1 AGCTTTAATAG-CAATTGTTTTGAAAACATTAGATAGAAATTTAAATTTTCTATTAACTT

PQ852120.1 AGCTTTAATAG-CAATTGTTTTGAAAACATTAGATAGAAATTTAAATTTTCTATTAACTT

PQ852121.1 AGCTTTAATAG-CAATTGTTTTGAAAACATTAGATAGAAATTTAAATTTTCTATTAACTT

PQ852123.1 AGCTTGATAAAGCAATTGTTTTGAAAACATTAAATAGAAACTAAAATTTTCTATTAACTT

PQ852116.1 AGCTTAAATAG-CAATTGTTTTGAAAACATTAGATAGAAATTAAAATTTTCTATTAACTT

PQ852117.1 AGCTTAAATAG-CAATTGTTTTGAAAACATTAGATAGAAATTAAAATTTTCTATTAACTT

PQ852115.1 AGCTTAAATAG-CAATTGTTTTGAAAACATTAGATAGAAACTAAAATTTTCTATTAACTT

PQ852124.1 AGCTTAAATAAGCAATTGTTTTGAAAACATTAGATAGAAACTAAAATTTTCTATTAACTT

***** *: *. ********************.******* *:**. *************

PQ868583.1 TACTAAATTTAATTATTATAATATAATTATTTTTAACCCAACAAATAAACAAATAAAACA

PQ868584.1 TACTAAATTTAATTATTATAATATAATTATTTTTAACCCAACAAATAAACAAATAAAACA

PQ852128.1 TACTAAAATTAATTATTATAATAAAATAATCTTTAATCCAATAAAAAAAATAATAAAACA

PQ852132.1 TACTAAAATTAATTATTATAATAAAATAATCTTTAATCCAATAAAAAAAATAATAAAACA

PQ852125.1 TACTAAAATTAATTATTATAATAAAATAATCTTTAACCCAATAAAAAAAATAATAAAACA

PQ852131.1 TACTAAAATTAATTATTATAATAAAATAATCTTTAATCCAATAAAAAAAATAATAAAACA

PQ852135.1 TACTAAAATTAATTATTATAATAAAATAATCTTTAATCCAATAAAAAAAATAATAAAACA

PQ852133.1 TACTAAAATTAATTATTATAATAAAATAATCTTTAATCCAATAAAAAAAATAATAAAACA

PQ852130.1 TACTAAAATTAATTATTATAATAAAATAATCTTTAATCCAATAAAAAAAATAATAAAACA

PQ852122.1 TACTAAATTTAATTATTATAATAACACTATCTTTAAACCAATAAATAAACAAATAAAACA

PQ868588.1 TACTAAATTTAATTATTATAATAACACTATCTTTAAACCAATAAATAAACAAATAAAACA

PQ868589.1 TACTAAATTTAATTATTATAATAACACTATCTTTAAACCAATAAATAAACAAATAAAACA

PQ852127.1 TACTAAAATTAATTATTATAATAAAATAATCTTTAATCCAATAAAAAAAATAATAAAACA

PQ852134.1 TACTAAAATTAATTATTATAATAAAATAATCTTTAATCCAATAAAAAAAATAATAAAACA

PQ852126.1 TACTAAAATTAATTATTATAATAAAATAATCTTTAATCCAATAAAAAAAATAATAAAACA

PQ815623.1 TACTAAATTTAATTATTATAATAAAAATATTTTTAATCCAACAAAAAATAAAATAAAACA

PQ868585.1 TACTAAATTTAATTATTATAATAAAATTATTTTAAAACCAACAAATAAACAAATAAAACA

PQ852118.1 TACTAAATTTAATTATTACAATAAAATTATCTTTAATCCAATAAATAAACAAATAAAACA

PQ868586.1 TACTAAATTTAATTATTACAATAAAATTATCTTTAATCCAATAAATAAACAAATAAAACA

PQ852119.1 TACTAAATTTAATTATTACAATAAAATTATCTTTAATCCAATAAATAAACAAATAAAACA

PQ868587.1 TACTATATTTAATTATTATAATAAAATTAATTTTAAACCAATAAACAAACTAATAAAACA

PQ852120.1 TACTATATTTAATTATTATAATAAAATTAATTTTAAACCAATAAACAAACTGATAAAACA

PQ852121.1 TACTATATTTAATTATTATAATAAAATTAATTTTAAACCAATAAACAAACTAATAAAACA

PQ852123.1 TACTAATTTTAATTATTATAATAAAATAATTTTTAATCCAATAAAAAAAATAATAAAACA

PQ852116.1 TACTAAATTTAATTATTATAATAGAAATATCTTTAAACCAATAAAAAAAATAATAAAACA

PQ852117.1 TACTAAATTTAATTATTATAATAAAAATATCTTTAAACCAATAAAAAAAATAATAAAACA

PQ852115.1 TACTAAATTTAATTATTATAATAAAATTATCTTTAAACCAATAAAAAAAATAATAAAACA

PQ852124.1 TACTAAATTTAATTATTATAATAAAAATATTTTCAATCCAATAAATATAAGAATAAAACA

*****:::********** **** .* :*: ** ** **** *** *::. .********

PQ868583.1 TAACGAAGCTGGTAAATATCT--

PQ868584.1 TAACGAAGCTGGTAAATATCT--

PQ852128.1 TAACGAAGCGGGTAA--------

PQ852132.1 TAACGAA----------------

PQ852125.1 TAACGAAGCGGGTAAA-------

PQ852131.1 TAACGAAGCGGGTAA--------

PQ852135.1 TAACGAA----------------

PQ852133.1 TAACGAAGCGGGT----------

PQ852130.1 TAACGAAGCGGGTAA--------

PQ852122.1 TAACGAA----------------

PQ868588.1 TAACGAA----------------

PQ868589.1 TAACGAA----------------

PQ852127.1 TAACGAAGCGGGTAA--------

PQ852134.1 TAACGAA----------------

PQ852126.1 TAACGAAGCGGGTAA--------

PQ815623.1 TAACGAAG---------------

PQ868585.1 TAACGAAGCGGGTAAA-------

PQ852118.1 TAACGAA----------------

PQ868586.1 TAACGAA----------------

PQ852119.1 TAACGAA----------------

PQ868587.1 TAACGA-----------------

PQ852120.1 TAACGAA----------------

PQ852121.1 TAACGAA----------------

PQ852123.1 TAACGAAGCTGGTAAATATCTTA

PQ852116.1 TAACGAA----------------

PQ852117.1 TAACGAA----------------

PQ852115.1 TAACGAAGCGGGTAA--------

PQ852124.1 TAACGAA----------------

**Sources of sand fly blood meals multiple sequence alignment** (according to GenBank accession numbers in Table 6)

A total 25 sequences of 25 blood meal sources: *Alectoris chukar* (PQ622922), *Bos taurus* (PQ852113), *Canis aureus* (PQ622917), *Canis lupus familiaris* (PQ622923), *Capra hircus* (PQ622921), *Columba livia* (PQ622926), *Equus asinus* (PQ622916*), Equus caballus* (PQ622919), *Equus hemionus* (PQ622939), *Erinaceus concolor* (PQ622925), *Felis catus* (PQ852112), *Gazella dorcas* (PQ622928), *Gazella gazella* (PQ622931), *Hemiechinus auratus* (PQ622924), *Homo sapiens* (PQ622920), *Hystrix indica* (PQ622934), *Lepus europaeus* (PQ622935), *Meles meles* (PQ622937), *Meriones tristrami* (PQ622918), *Mus musculus* (PQ622938), *Ovis aries* (PQ852114), *Procavia capensis* (PQ622930), *Psammomys obesus* (PQ622940), *Sus scrofa* (PQ622929), *Vulpes vulpes* (PQ622927).

PQ622923.1 ----------------------------------------------TTAACTTAACTAAA

PQ622925.1 -------------------------------CTGATAATTAATGTTATAGTGATAATAAT

PQ852112.1 ---CCCGTCACCCTCCTCAAG--TGGTAACTCCCAAAAAAACCTATTTAAATTATCACAC

PQ622937.1 ---CCCGTCACCCTCCTCAAG--CAACATACCCAAGCATTACATAATAAAACAATATTAA

PQ622916.1 -----------------------------------------------CCGTGACCCAAAC

PQ622940.1 ---------------------------------------------------ATACATATT

PQ622921.1 -------------------------------------CAGCCTATTAACACGCATCAACT

PQ852114.1 CCGCCCGTCACCCTCCTCAAGTAAATATGATATACTTAAACCTATTTACATATATCAACC

PQ852113.1 --GCCCGTCACCCTCCTCAAATAGATTCAGTGCATCTAACCCTATTTAAACGCACTAGCT

PQ622931.1 ------------------------------TATAAAATAACCTATATTCCATTACACAAC

PQ622919.1 ---------------------------------------ACATAAAACCGTGACCCAAAC

PQ622920.1 ------------------------------------TTAACTAAAACCCCTACGCTTTAT

PQ622917.1 ------------------------------------------------------------

PQ622929.1 ---------------------------GTATAAAATAACCCTATATTCCATTACACAACC

PQ622924.1 ----------------------ATAAATATATACATTAATTATATTACAACGATAATAGT

PQ622934.1 ------------------------------------------------------------

PQ622939.1 --------------------------------ATATAACACAAAACCCGTGACCCTAAAC

PQ622928.1 ----------------------------------CTTAATATATTTTAAACGCACTAACC

PQ622930.1 ------------------------------------------------------------

PQ622935.1 ------------------------------------------------------------

PQ622918.1 ------------------------------------------------------------

PQ622938.1 ------------------------------------------------------------

PQ622927.1 ------------------------------------------------------------

PQ622922.1 -------------------------------------------AAATAATACCCGCCCCG

PQ622926.1 ------------------------------------------------------------

PQ622923.1 ACACAAGAGGAGACAAGTCGTAACAAGGTAAGCATACCGG-AAGGTGTGCTTGGATTAAT

PQ622925.1 ATACAAGAAGAGATAAGTCGTAACAAGGTAAGTATACTGG-AAAGTGTACTTGGACTATC

PQ852112.1 CCACAAGAGGAGATAAGTCGTAACAAGGTAAGCATACTGG-AAAGTGTGCTTGG--ATAA

PQ622937.1 G-ACAAGAGGAGATAAGTCGTAACAAGGTAAGCATACTGG-AAAGTGTGCTTGGGTAAAT

PQ622916.1 ATATGAAAGGAGACAAGTCGTAACAAGGTAAGTATACCGG-AAGGTGTACTTG-GATAAC

PQ622940.1 ATAATGAGAGAGGTAAGTCGTAACAAGGTAAGCATACTGG-AAAGTGTGCTTGGATCAAC

PQ622921.1 ACATGAGAGGAGATAAGTCGTAACAAGGTAAGCATACCTGGAAAGTGTGCTTGGATAAAT

PQ852114.1 ACACGAGAGGAGACAAGTCGTAACAAGGTAAGCATACTGG-AAAGTGTGCTTGGATAAAC

PQ852113.1 ACATGAGAGGAGACAAGTCGTAACAAGGTAAGCATACTGG-AAAGTGTGCTTGGATAAAT

PQ622931.1 CATGCAAGAGAGACAAGTCGTAACAAGGTAAGCATACTGG-AAAGTGTGCTTGGATTACC

PQ622919.1 ATATGAAAGGAGACAAGTCGTAACAAGGTAAGTATACCGG-AAGGTGTACTTGGAT-AAC

PQ622920.1 ATAG---AGGAGACAAGTCGTAACATGGTAAGTGTACTGG-AAAGTGCACTTGGACGAAC

PQ622917.1 ----------------GTCGTAACAAGGTAAGCATACCGG-AAGGGGTGCTTGGATTAAT

PQ622929.1 ATGCAAGAAGAGACAAGTCGTAACAAGGTAAGCATACTGG-AAAGTGTGCTTGGATTACC

PQ622924.1 TTATAAGAAGAGATAAGTCGTAACAAGGTAAGTATACTGG-AAAGTGTACCTTGGACAAT

PQ622934.1 ----------------GTCGTAACAAGGTAAGCATACTGG-AAAGTGTGCTTGG-ATAAA

PQ622939.1 ATATGAAAGGAGACAAGTCGTAACAAGGTAAGTATACCGG-AAGGGGTACTTGG-ATAAC

PQ622928.1 ACATGAGAGGAGACAAGTCGTAACAAGGTAAGCATACTGG-AAAGTGTGCTTG-GATAAA

PQ622930.1 ----------------GTCGTAACAAGGTAAGCATACCTGGAAAGTGTGCTTGGAAAAAC

PQ622935.1 ----------------GTCGTAACAAGGTAAGCGTACTGG-AAAGTGTGCTTGGATCAAC

PQ622918.1 ----------AGGAAAGTCGTAACAAGGTAAGCGTACCTGGAAAGTGTGCTTGGATCAAC

PQ622938.1 ----------------GTCGTAACAAGGTAAGCATACTGG-AAAGTGTGCTTGGAATAAT

PQ622927.1 ----------------GTCGTAACAAGGTAAGCATACCGG-AAGGTGTGCTTGGATTAAC

PQ622922.1 GCTAAAGACGAGGTAAGTCGTAACAAGGTAAGTGTACCGGAAGGTGCACTTTAG-ACCAC

PQ622926.1 -----------GGTAAGTCGTAACAAGGTAAGTGTACCGGAAGGGGCACTTTGGCACACC

*********:****** .*** * *.. *

PQ622923.1 CAAAGTGTAGCTTAACTAAAG--CGTCTGGCCTACACCCAG---AAGATTTCATTACTTA

PQ622925.1 GGAGTGTAGCTTAAGATAAAG--CGACTGCCTTACACCCAG---GA-AATTCTAATTATA

PQ852112.1 CAAGATGTAGCTTAAACAAAG--CATCTGGCTTACACCCAG---AAGATTTCATATTAAA

PQ622937.1 CAAAGTGTAGCTTAACTAAAG--CGCCTGGCTTACACCCAG---AGGATTTCATACATCA

PQ622916.1 CAAAGTGTAGCTTAAACAAAG--CATCCAGCTTACACCTAG---AAGATTTCACTCAGAG

PQ622940.1 CAAAGTGTAGCTTAATAAAAAG-CATCTGGCTTACACCCGG---AAGAATTCACCAT-TA

PQ622921.1 CAAGATATAGCTTAACCAAAG--CACCTAGTTTACACCTAG---AAGATTTCACATATTA

PQ852114.1 CAAGATATAGCTTAATTAAAG--CATCTAGTTTACACCTAG---AAGATTTCACACATTA

PQ852113.1 CAAGATATAGCTTAAACAAAG--CATCCAGTTTACACCTAG---AAGACTTCATTCATTA

PQ622931.1 AAAGCATAGCTTAAACTAAAG--CACCTAGTTTACACCTAG---AAGATCCCACAATGTA

PQ622919.1 CAAAGTGTAGCTTAAACAAAG--CATCCAGCTTACACCTAG---AAGATTTCACTCAAAA

PQ622920.1 CAGAGTGTAGCTTAACACAAAG-CACCCAACTTACACTTAG---GAGATTTCAACTTAAC

PQ622917.1 CAAAGTGTAGCTTAACTAAAG--CGTCTGGCCTACACCCAG---AAGATTTCAT-TACTT

PQ622929.1 AAAGCATAGCTTAAACTAAAG--CACCTAGTTTACACCTAG---AAGATCCCAC-AATGT

PQ622924.1 CGGAGTGTAGCTTAAAATAAAG-CGACTGCTTTACACCCAG---GAGATTCTA--CCATT

PQ622934.1 CAAAGTGTAGCTTAAATAAAG--CATCTGGCTTACACCCAG---AAGACTTCAGGTAATA

PQ622939.1 CAAAGTGTAGCTTAAATAAAG--CATCCAGCTTACACCTAG---AAGATTTCAT-TCAAA

PQ622928.1 CCAAGACATAGCTTAAACTAAAGCATCTAGTTTACACCTAG---AAGATTCCATACACCA

PQ622930.1 TCAAAGTGTAGCTTAACACAAAGCACCTGGCCTACACCCAG---AAGATTTCAACCCCAT

PQ622935.1 CAAAGCGTAGCTATACCACAAGGCATCTGGCCTTACACCCAGAAGGAATTTCATAATAGG

PQ622918.1 CAAAGCGTAG-CATACCACAAAGCATCTGGCCTACACCCAG--AAGGAATTCATATTCG-

PQ622938.1 CATAGTGTAGCTTAATATTAAAGCATCTGGCCTACACCCAG---AAGATTTCATGACCAA

PQ622927.1 CAAAGTGTAGCTTAATA--AAAGCATCTGGCTTACACCCAG---AAGATTTCATGATTAA

PQ622922.1 CAAGGCGTAGCTATAAACCAAAGCACTCAGCTTACACCTGA---AAGATGCCT-TCAAAG

PQ622926.1 CAAGACGTAGCTACAATGTAAAGCATTCAGCTTACACCTGA---AAGATGCCTGCCACAC

. : . *. *. . *:... . .. * :

PQ622923.1 TG-GCCACTTTG-------AACAAAAGCTAGCCCAACTAACCCCAAACTTA---AGTATT

PQ622925.1 TG-GACACTTCG-------AACTAATTCTAGCCTACTAAATTATAACTACT---TATTAG

PQ852112.1 CTGACCATCTTG-------AGCTAAAGCTAGCCCAAACATCTACAAACACA---ACTAAC

PQ622937.1 TG-ACCACTTTG-------AACCAAAACTAGCCCAACCAACTACCAACTCA---ATTACT

PQ622916.1 TG-AACACTTTG-------AACTAAAGCTAGCCCAAACAATACCCAACTCA---ACT-AC

PQ622940.1 TG-AACACTTTG-------AGCCAAACCTAGCCCTATCAAGAAAAAAAAAT---AATGAT

PQ622921.1 TG-AATATCTTG-------AACTATATCTAGCCCAATCCCCCCCCCAT-----CTAAATT

PQ852114.1 TG-AGTATCTTG-------AACTATACCTAGCCCAAAACCTCCCACTCTCCAGTTTAAAT

PQ852113.1 TG-AATATCTTG-------AACTAGACCTAGCCCAAAGATACCCTCTCG---ACTAAACA

PQ622931.1 TG-GGTACTTTG-------AACCAAAGCTAGCTCAACATACTAAACAAATACAAAAATAC

PQ622919.1 TG-AACACTTTG-------AACTAAAGCTAGCCCAAACAATACCTAATT------CAATT

PQ622920.1 TTGACCGCTCTG-------AGCTAAACCTAGCCCCAAACCCACTCCACC------TTACT

PQ622917.1 ATGACCACTTTG-------AACAAAAGCTAGCCCAACTAACC-CCAAACT----TAAGTA

PQ622929.1 ATGGGTACTTTG-------AACCAAAGCTAGCTCAACATACT-AAACAAA----TACAAA

PQ622924.1 ATGGACACTTCG-------AACTAAAGCTAGCCTATTAAAAATATAATAT----CAAATA

PQ622934.1 CTGGCCACTTTG-------AACCAATTCTAGCCCAAATATACTACTAGTA----AAACTA

PQ622939.1 ATGAACACTTTG-------AACTAAAGCTAGCCCAAACAACG-TCCAACT----CAACTA

PQ622928.1 TGAATGTCTTG--------AACTATACCTAGCCCAAGTTTTTACCACAAAC--CTAATAA

PQ622930.1 TGACCACTTTG--------AACTAAAACTAGCCTGTCCACTCGACAAACCA--AACCACC

PQ622935.1 TGAACACTTTTGA------CCCAGAACCTAGCTCTAC--CAAAGAAAAAAC--CAACCTA

PQ622918.1 TGAACACTTTTGA------CCCAG-ACCTAGCTCTAC--CAAAG-AAAAAC--ACACCTC

PQ622938.1 TGAACACTCTG--------AACTAATTCTAGCCCTAG--CCCTACACAAAT--ATAATTA

PQ622927.1 TGACCACTTTG--------AACGAAAGCTAGCCCAAT--CGACTCTAAATT--AAACTAT

PQ622922.1 ATAAGGTCGCCTTGATCTGCCCTCTCTCTAGCCCAATCAACCTCACCCTAAATATCAAAA

PQ622926.1 ACCAGGTCGTCTTGAAG----CCAACTCTAGCCCAACCCCCCACGACAACCGCACATAAA

* *****

PQ622923.1 ACAG-ACACAT-AAAATAAAACATTTAGTTAAACAATAAAAGTATAGGAGATAGAAATTT

PQ622925.1 CCTATTAAATT-TAATTAAAACATTCATTTAAAATTTACCAGTATAGGAGATAGAAAATT

PQ852112.1 ACTAGAAAAT--AAAACAAAACATTTAGTCACCTTATAAAAGTATAGGAGATAGAAATTT

PQ622937.1 ACAATAAATCTTCAATCAAAACATTTAGTACTACGATTATAGTATAGGAGATAGAAATTC

PQ622916.1 CCTTAGTCACT-TAACTAAAACATTCACCAAACCATTAAAG-TATAGGAGATAGAAATTT

PQ622940.1 ATTTACTTCAT-AAATCAAATCATTCAATCTAATTAAAGTA-TTAGAGAAAGAAATTCTT

PQ622921.1 ACCAAAACAGGCTAAAACAAAACATT--TACCCCAATTAA--------------------

PQ852114.1 AACTAAATTAATTAAAATAAAACATT--TACCCTAATTAAAGTATAGGAGATAGAAATTC

PQ852113.1 ACCAAGATAGAATAAAACAAAACATTTAATCCCAATTTAAAGTATAGGAGATAG-AAATC

PQ622931.1 ACCAAAATAAAATAAAACATTCACCT------AACATTAAAGTATAGGAGATAGAAATTT

PQ622919.1 ACCCTTAGTCACTTAACTAAAACATTCACCAAACCATTAAAGTATAGGAGATAGAAATTT

PQ622920.1 ACCAGACAACCTTAGCCAAACCATTTACCCAAAT----AAAGTATAGGCGATAGAAATTG

PQ622917.1 TCACAGACATATAAAGTAAAACATTTAGTTAAATAATAAAAGTATAGGAGATAGAAATTT

PQ622929.1 AATACACCAAAATAAAATAAAACATTCACCTAACATTAAAG-TATAGGAGATAGAAATTT

PQ622924.1 TAATTATTAATTTAATTAAAGCATTTACTACCAATATACTAGTATAGGCGATAGAAAATT

PQ622934.1 TTTTCATACATTAAAACAAAACATTCATACCACT----AAAGTATAGGCGATAGAAATTT

PQ622939.1 CCCTTAGTCACTTAACTAAAACATTCACCAAACCATT-AAAGTATAGGAGATAGAAATTT

PQ622928.1 TTAAAATAAAATTAATTAAAACATTCACCCCCTGGATTAAAG-TATAGGAGATAGAAATT

PQ622930.1 CTAAAAATATTAAAATAAAACATTCAACCCTTAACCTTAAAAGTATAGGAGATAGAAAGT

PQ622935.1 CGACTCTAATGTAACCCAATTCATTTAGCCTATTTAAGGTTT-TGGGGAAGGAATTTCTC

PQ622918.1 CGACTCTAATGTAACCCAAATCATTTAGTCTATTTAAGGTAT-TGGGGAAAGAAATTCTC

PQ622938.1 T-ACTATTATATAAATCAAAACATTTATCCTACTAAAAGTAT-TGGAGAAAGAAATTCGT

PQ622927.1 C-ACAGAGTTATAAAACAAAACATTTAGTCAAACCATTAAAG-TATAGGAG-ATAGAAAT

PQ622922.1 ATTCACTTCACACACTCAACCAAAACATTCTAACCGTCCAAGTATAGGTGATAGAAAAGA

PQ622926.1 AATCTACTAAATCTTTAAATCAAAACATTCTT-CCAACTAAGTATAGGCGATAGAAAAGT

: * .

PQ622923.1 TAATTGG--AGCGATAGAGAT----AGTACCGTAAGGGAATG-ATG-AAAG-------AC

PQ622925.1 CAATATAG-CGCGATAGAAAA----AGTACCGTAAGGGAACG-ATTGAAAG-------AG

PQ852112.1 AACTTGG--CGCTATAGAGAA----AGTACCGCAAGGGAAAG-ATGAAAGA-------TA

PQ622937.1 TACTTGG--AGCTATAGAGGA----AGTACCGTAAGGGAATG-ATGAAAG---------A

PQ622916.1 TAACTTGG-CGCTATAGAGAA----AGTACCGTAAGG-GAAC-GATGAAAG-------AT

PQ622940.1 TTTTTAGA-AGCTATAGAAGA----AGTACCGCAGGG-AAAG-ATGAAAGA-------AC

PQ622921.1 ------------------------------------------------------------

PQ852114.1 TAAACACGGCGCTATAGAGAA----AGTACCGCAAGGGAATG-ATGAAAGA------AAA

PQ852113.1 TAAGTACGGCGCTATAGAGAA----AGTACCGCAAGGGAACG-ATGAAAGA------AAA

PQ622931.1 TTATCCTGACGCTATAGAGAT----AGTACCGTAAGGGAAAG-ATGAAAG-------AAT

PQ622919.1 -TAACTTGGCGCTATAGAGAA----AGTACCGTAAGGGAACG-ATGAAAG--------AT

PQ622920.1 -AAACCTGGCGCAATAGATAT----AGTACCGCAAGGGAAAG-ATGAAAA--------AT

PQ622917.1 TAATTGG--AGCGATAGAGAT----AGTACCGTAAGGGAATG-ATGAAAG--------AC

PQ622929.1 TTATCCTGACGCTATAGAGAT----AGTACCGTAAGGGAAAG-ATGAAAGA-------AT

PQ622924.1 TATATAG--CGCAATAGATAA----AGTACCGTAAGGGAACG-ATTGAAA--------GA

PQ622934.1 TATTAGG--CGCTATAGAGAA----AGTACCGCAAGGGAAAG-ATGAAAG--------AA

PQ622939.1 TAACCTGG-CGCTATAGAGAA----AGTACCGTAAGGGAACG-ATGAAAG--------AT

PQ622928.1 CTAAAATGGCGCAATAGAGAA----AGTACCGCAAGGGAATG-ATGAAAGA------AAA

PQ622930.1 TTACTCAG-CGCCATAGAGAC----AGTACCGCAAGGGAAAG-ATGAAAGA------TAA

PQ622935.1 TTCTAGAAGG-CAATAGAAAC----AGTACCGCAGGGGAAAAGATGAAAGA------CCT

PQ622918.1 TTCTAGAAG--CTATAGAAAC----AGTACCGCAGGG-AAAAGATGAAAGA------CCT

PQ622938.1 ACATCTAGGAGCTATAGAACT----AGTACCGCAAGGGAAAG-ATGAAAGA------CTA

PQ622927.1 TCTAATTGGAGCTATAGAGAT----AGTACCGCAAGGGAAATGATGAAAGA------CAT

PQ622922.1 CCCTCCGG-CGCAATAGAGATTAGCTGTACCGTAGGGGAAAAGATGAAATAACATGAAAT

PQ622926.1 CATTAGG--CGCTATAGAGAT---CTGTACCGCAAGGGAGAG-ATGAAATAACAA-TGAA

PQ622923.1 ATCTTAACAGTATTAAACAGCAAAGATTACCCCTTCTACCTTTTGCATAATGAACTAGCC

PQ622925.1 TAATTGAAAGTAAAAAATAGCAAAGATTAAACCTTGTACCTTTTGCATAATGATTTAGCC

PQ852112.1 AAATTAAAAGCACCACACAGCAAAGATTACCCCTTGTACCTTTTGCATAATGAGTTAGCT

PQ622937.1 AACTTTAAAGTAATAAATAGCAAAGATTACCCCTTGTACCTTTTGCATAATGAATTAGCC

PQ622916.1 GTATTAAAAGTACTAAACAGCAAAGCTTACCCCTTTTACCTTTTGCATAATGATTTAACT

PQ622940.1 TAGTTCATAGTAAAAATAAGCAAAGATTTCACCTTGTACCTTTTGCATTATGAGTCAATT

PQ622921.1 ------------------------------------------------------------

PQ852114.1 AAAATCACAGTACAAAAAAGCAAAGATTAACCCTTGTACCTTTTGCATAATGAATTAACG

PQ852113.1 AAACTAAAAGTATAAAAAAGCAAAGATTACCCCTTGTACCTTTTGCATAATGAATTAACT

PQ622931.1 ACAATAAAAGTAAAAAAAAGCAAAGATTACCCCTTCTACCTTTTGCTTAATGGTTTACCA

PQ622919.1 GCATTAAAAGTACTAAACAGCAAAGCTTACCCCTTTTACCTTTTGCATAATGATTTAACT

PQ622920.1 TATAACCAAGCATAATATAGCAAGGACTAACCCCTATACCTTCTGCATAATGAATTAACT

PQ622917.1 ATCTTAACAGTATTAAACAGCAAAGATTACCCCTTTTACCTTTTGCATAATGAACTAGCC

PQ622929.1 AAAATAAAAGTAAAAAAAAGCAAAGATTACCCCTTCTACCTTTTGCATAATGGTTTAACC

PQ622924.1 TAATTTAAAGTAATAAATAGCAAAGATTAAATCTTTTACCTTTTGCATAATGATTTAGCC

PQ622934.1 TTATTAAAAGTGTAAAACAGCATAGATTAATACTACTACCTTTTGCATCATGAGCTAACT

PQ622939.1 GCATTAAAAGTACTAAACAGCAAAGCTTACCCCTTTTACCTTTTGCATAATGATTTAACC

PQ622928.1 ACTATCAAAGTACAAAAAAGCAAAGATTACCCCTTGTACCTTTTGCATAATGAATTAACT

PQ622930.1 CTTG--ATAGTACAAAACTGCAAAGATTAACCCCTGTACCTTTTGCATAATGAATTAACT

PQ622935.1 CACCTTATGGTAAAACCAGGCAAGGATTCCCCCTTGTCCCTTTTGCATTATGGATCATTT

PQ622918.1 CACCTTATAGTAAAAACAGGCAAGGATTCCCCCTTGTCCCTTTTGCATTATGGATCATTT

PQ622938.1 ATTAAAAG--TAAGAACAAGCAAAGATTAAACCTTGTACCTTTTGCATAATGAACTAACT

PQ622927.1 TTTCAAAG--TACAAAACAGCAAAGATTACACCTTGTACCTTTTGCATAATGAGCTAGCT

PQ622922.1 CAACTATAAGCAAGAAACAGTAAAGACCAGCCCTTGTACCTCTTGCATCATGATTTAGCG

PQ622926.1 CTAAGCCGAGCAATAAACAGCAAAGATAAACCCTTGTACCTCTTGCATTATGGTCTAGCT

PQ622923.1 AGAAACAACTTAA-CAAAGAGAACTTAAGCTAAGCTCCCCGAAACCAGA-CGAG--CTAC

PQ622925.1 AGAAAAAAATTAA-CAAAGAGAACTAAAGCTAATTACCCCGAAACCAGA-CGAG--CTAT

PQ852112.1 AG-AACAACCTAA-CAAAGAGAACTTCAGCTAGGCCCCCCGAAACCAGA-CGAG--CTAC

PQ622937.1 AG-AATAATTTAG-CAAAGAGACCTTAAGCTAACCTTCCCGAAACCAGA-CGAG--CTAC

PQ622916.1 AGAATAAACTTAG-CAAAGAGAACTTAAGCTAAGCACCCCGAAACCAGA-CGAG--CTAC

PQ622940.1 AGAACCCTAACTAGCTAAGAGAACTTCAGCTAGGGCCCCCGAAACCAAA-CGAG--CTAC

PQ622921.1 ------------------------------------------------------------

PQ852114.1 AGCAAAAAACTTAACAAAACGAATTTTAGCTAAGTAACCCGAAACCAGA-CGAG--CTAC

PQ852113.1 AGTATAAGACTTAACAAAATGAATTTTAGCTAAGCAGCCCGAAACCAGA-CGAG--CTAC

PQ622931.1 GGAAAAAATCTAACAAAGGGGACTTTTAGCTAGTTCCCCCGGAACCCGG-GGGGGCCTAC

PQ622919.1 AG-AATAAACTTAGCAAAGAGAACTTAAGCTAAGCACCCCGAAACCAGA-CGAG--CTAC

PQ622920.1 AG-AAATAACTTTGCAAGGAGAGCCAAAGCTAAGACCCCCGAAACCAGA-CGAG--CTAC

PQ622917.1 AG-AAACGACTTAACAAAGAGAACTTAAGCTAAGCTCCCCGAAACCAGA-CGAG--CTAC

PQ622929.1 AG-AAAAAATCTAACAAAGAGAACTTTAGCTAGATACCCCGAAACCAGA-CGAG--CTAC

PQ622924.1 AG-AATAAAATTAGCAAAGAGAACTTAAGCTAATTACCCCGAAACCAGA-CGAG--CTAT

PQ622934.1 AG-AACTCCCTTTGCAAAAAGAATTTAAGCAAAGACCCCCGAAACCAAA-CGAG--CTAC

PQ622939.1 AG-AATAAACTTAGCAAAGAGAACTTAAGCTAAGCACCCCGAAACCAGA-CGAG--CTAC

PQ622928.1 AGTAAAAACTTAA-CAAAATGAATTTTAGCTAAGTAACCCGAAACCAGA-CGAG--CTAC

PQ622930.1 AGAACAAGCCTGA-CAAAGAGAACTGTAGCCAGGAACCCCGAAACCGGA-CGAG--CTAC

PQ622935.1 AGATTTTTCCCAAGCTAGGAGACCTTCAGTTAGCACCCCCGAACCCAAA-CGAG--CTAC

PQ622918.1 AGAATTTTCCCTAGCTAAGAGAACTTCAGTTAGCGCCCCCGAACCCAAA-CGAG--CTAC

PQ622938.1 AGAAAACTTCTAA-CTAAAAGAATTACAGCTAGAAACCCCGAAACCAAA-CGAG--CTAC

PQ622927.1 AGAAATAACTTAA-CAAAGAGAACTTAAGCTAAGTACCCCGAAACCAGA-CGAG--CTAC

PQ622922.1 AG--AACAACCAAGCAAAGCGAACTAAAGTTTGCCCCCCCGAAACCCAAGCGAG--CTAC

PQ622926.1 AG--AATAACCAAGCAAAGTGGACTTAAGCTTGCCACCCCGAAACCTAAGCGAG--CTAC

PQ622923.1 CCATAAACAA--TCTAAAAGGATCAACTCATCTATGTAGCAAAATAGTGAGAAGATTTGT

PQ622925.1 CTAATAATAA--CTTTCTG-AGTTAACTCATCTATGTGGCAAAATAGTGAGAAGATTACT

PQ852112.1 CCATGAACAA--TCTATTACAGGATGAACTC-----------------------------

PQ622937.1 CTATAAACAA--TCCACAG--GGATAAACT------------------------------

PQ622916.1 CTATGAACAG--TTATAAAGAACCAACTCATCTATGTCGCAAAATAGTGAGAAGATTCGT

PQ622940.1 CCTAAAGCAA--CTTTATG-AGTTAACCCGTCCATGTGGCAAAATGGTGGGAAGATTTTA

PQ622921.1 ------------------------------------------------------------

PQ852114.1 TTATAGACAGT-TTATTAG-AACCAACTC-------------------------------

PQ852113.1 TCACAAACAGT-TTACCAAGAACTAACTC-------------------------------

PQ622931.1 CCATGGGGCGG-TTTTTA------------------------------------------

PQ622919.1 CTATGAACAG--TTACAAAGAACCAACTCATCTATGTCGCAAAATAGTGAGAAGATTCGT

PQ622920.1 CTAAGAACAG--CTAAAAG-AGCACACCCGTCTATGTAGCAAAATAGTGGGAAGATTTAT

PQ622917.1 CCATAAACAA--TCTAAAAGGATCAACTCATCTATGTAGCAAAATAGTGAGAAGATTTGT

PQ622929.1 CCATGAGCAG--TTTAAAAGAACCAACTCATCTATGTGGCAAAATAGTGAGAAGACTTGT

PQ622924.1 CTAACAACAA--CTTTATG-AGTCAACTCATCTATGTGACAAAATAGTGAGAAGATTGCT

PQ622934.1 TTTCAGACAG--CTAAATTGAGCCAACCCGTCTATGTGGCAAAATAGTGGGAAGATCTGA

PQ622939.1 CTACGAACAG--TTACAAAGAACCAACTCATCTATGTCGCAAAATAGTGAGAAGATTCAT

PQ622928.1 TTATGAACAG--TTATTAAGAACCAACTCATCTATGTAGCAAAATAGTGAGAAGATTTAT

PQ622930.1 CTATGAGCAG--TTTACAG-AACCAACCCGTCTATGTAGCAAAATAGTGGGAAGACTTGT

PQ622935.1 CTAGGACCAC--CTTTTAT-GAGTTAC---------------------------------

PQ622918.1 CTAAGACCAA--CTTTATG-AGTTACCCCGTCCATGTGGCAGATGG-TGGGAGGATTTTT

PQ622938.1 CTAAAAACAA--TTTTATG-AATCAACTCGTCTATGTGGCAAAATAGTGAGAAGATTTTT

PQ622927.1 CTATGAACAA--TCTAAAAGGATCAACTCGTCTATGTGGCAAAATAGTGAGAAGATTTAT

PQ622922.1 TTGCAAGCAGCTAATAATTGAGCGAACCCGTCTCTGTTGCAAAAGAGTGGGACGACTTGC

PQ622926.1 TTGTAAGCAGCTAACCCCTGAGCGAACCCGTCTCTGTTGCAAAAGAGTGGGACGACTTAC

PQ622923.1 GGGTAGAGGTGAAAAGCCTAACGAGCCTTGTGATAGCTTGGTT-----------------

PQ622925.1 AGATAGAGGTGAAATGCCTATCGAGC----------------------------------

PQ852112.1 ------------------------------------------------------------

PQ622937.1 ------------------------------------------------------------

PQ622916.1 AGGTAGAGGTGAAAAGCCCAACGAGC----------------------------------

PQ622940.1 AGGTAGAGGTGAAAAGCCTAA---------------------------------------

PQ622921.1 ------------------------------------------------------------

PQ852114.1 ------------------------------------------------------------

PQ852113.1 ------------------------------------------------------------

PQ622931.1 ------------------------------------------------------------

PQ622919.1 AGGTAGAGGTGAAAAGCCCAACGAGCCTTGTGAT--------------------------

PQ622920.1 AGGTAGAGGCGACAAACCTACCGAGC----------------------------------

PQ622917.1 GGGTAGAGGTGAAAAGCCTAACGAGCCTTGTGATAGCTGGTTAGGCTTTTCACCTCTACC

PQ622929.1 AGGTAGAGGTGAAAAGCCTAACGAGC----------------------------------

PQ622924.1 AGATAGAGGTGAAAAGCCTATCGAGC----------------------------------

PQ622934.1 AAGTAGAGGTGAAAAGCCAACCGAGC----------------------------------

PQ622939.1 AGGTAGAGGTGAAAAGCCCAACGAGC----------------------------------

PQ622928.1 AAGTAGAGGTGAAACGCCTAACGAGC----------------------------------

PQ622930.1 AGGTAGAGGTGAAAAGCCAACCGAGC----------------------------------

PQ622935.1 ------------------------------------------------------------

PQ622918.1 AGGTAGAGGTGAAAAGCCTAACGAGCCTGGTGATAGCTGGGTTAGGCTTTTCACCTCTAC

PQ622938.1 AGGTAGAGGTGAAAAGCCTAACGAGC----------------------------------

PQ622927.1 AGGTAGAGGTGAAAAGCCAAACGAGC----------------------------------

PQ622922.1 CAGTAGAGGTGAAAAGCCAACCGAGCCTTGTGAT--------------------------

PQ622926.1 TAGTAGAGGTGAAAAGCCAATCGAGCCTTGTGATAGCTGGTTACAGTAGCTCGCTTAGGT

PQ622923.1 ---------

PQ622925.1 ---------

PQ852112.1 ---------

PQ622937.1 ---------

PQ622916.1 ---------

PQ622940.1 ---------

PQ622921.1 ---------

PQ852114.1 ---------

PQ852113.1 ---------

PQ622931.1 ---------

PQ622919.1 ---------

PQ622920.1 ---------

PQ622917.1 ---------

PQ622929.1 ---------

PQ622924.1 ---------

PQ622934.1 ---------

PQ622939.1 ---------

PQ622928.1 ---------

PQ622930.1 ---------

PQ622935.1 ---------

PQ622918.1 CTAAAAAT-

PQ622938.1 ---------

PQ622927.1 ---------

PQ622922.1 ---------

PQ622926.1 TTCGGGGTG
